# The roles of HD-ZIP proteins in plant abiotic stress tolerance

**DOI:** 10.3389/fpls.2022.1027071

**Published:** 2022-10-12

**Authors:** Yuxia Li, Zongran Yang, Yuanyuan Zhang, Jinjiao Guo, Lili Liu, Chengfeng Wang, Baoshan Wang, Guoliang Han

**Affiliations:** Shandong Provincial Key Laboratory of Plant Stress Research, College of Life Sciences, Shandong Normal University, Shandong, China

**Keywords:** HD-ZIP protein, transcription factor, abiotic stress, plant growth and development, molecular mechanism

## Abstract

Homeodomain leucine zipper (HD-ZIP) proteins are plant-specific transcription factors that contain a homeodomain (HD) and a leucine zipper (LZ) domain. The highly conserved HD binds specifically to DNA and the LZ mediates homodimer or heterodimer formation. HD-ZIP transcription factors control plant growth, development, and responses to abiotic stress by regulating downstream target genes and hormone regulatory pathways. HD-ZIP proteins are divided into four subclasses (I–IV) according to their sequence conservation and function. The genome-wide identification and expression profile analysis of HD-ZIP proteins in model plants such as Arabidopsis (*Arabidopsis thaliana*) and rice (*Oryza sativa*) have improved our understanding of the functions of the different subclasses. In this review, we mainly summarize and discuss the roles of HD-ZIP proteins in plant response to abiotic stresses such as drought, salinity, low temperature, and harmful metals. HD-ZIP proteins mainly mediate plant stress tolerance by regulating the expression of downstream stress-related genes through abscisic acid (ABA) mediated signaling pathways, and also by regulating plant growth and development. This review provides a basis for understanding the roles of HD-ZIP proteins and potential targets for breeding abiotic stress tolerance in plants.

## Introduction

Common abiotic stresses include drought, high and low temperatures, oxidative stress, salinity, and toxic metals, and both abiotic and biotic stresses reduce crop yields, resulting in significant economic losses (Patel et al., 2017). Globally, approximately 20% and 22% of arable land is affected by drought and salinization, reducing crop yields in affected areas by more than 50% (Vinocur and Altman, 2005; Agarwal et al., 2017). Harmful metals exist as free ions or with functional groups (such as glutathione sulfhydryl groups), and their reduction produces reactive oxygen species (ROS) that ultimately lead to oxidative damage to plant cells (Kaur et al., 2021). Plants have evolved different mechanisms to deal with abiotic stresses (Sytar et al., 2021). These adaptive mechanisms involve changes in plant physiology and biochemistry, molecular and cellular levels, and are regulated by complex networks. Given the impacts of climate change and severe weather, as well as soil contamination with salt and metals, on crop production, it is important to understand the ability of plants to respond to single and combined stresses to improve crop resilience (Baillo et al., 2019).

Transcription factors (TFs) are involved in regulatory networks that regulate plant growth and development and respond to stress (Haak et al., 2017). TFs activate or repress the transcription of these target genes by binding to specific *cis*-acting elements within the promoters of these target genes, and participate in signal transduction pathways. In plants, about 10% of genes encode transcription factors that perform their specific functions during different growth and developmental stages of plants. Transcription factors are divided into different families according to their DNA-binding domains. Arabidopsis (*Arabidopsis thaliana*) has an estimated 30 transcription factor families, including 1922 transcription factors (Aglawe et al., 2012). Several types of TFs involved in abiotic stress have been cloned, including members of the Homeodomain-leucine zipper (HD-ZIP) and APETALA2, WRKY (it is characterized by a highly conserved WRKYGQK core motif at the N-terminus), NAM, ATAF and CUC (NAC), Ethylene-responsive element binding proteins (AP2/EREBP), v-myb avian myeloblastosis viral oncogene homolog (MYB), heat stress transcription factor (HSF), zinc finger proteins (ZFPs), and basic helix-loop-helix (bHLH) transcription factor families (Wang et al., 2016; Li et al., 2019e). Since transcription factors are major regulators of stress-related genes, they can be used for genetic engineering to improve stress tolerance in plants. Clustered regularly interspaced short palindromic repeats (CRISPR)/CRISPR-associated protein 9 (Cas9) gene editing technology is an important tool used to study TFs to improve plant stress resistance. Researchers comprehensively reviewed the use of CRISPR/Cas9 to evaluate the roles of genes in environmental stress tolerance, transcriptional and translational regulation, and crop improvement (Bao et al., 2019). Many TFs respond to stress and regulate a large number of downstream genes; therefore, regulating the expression of transcription factor genes is a research hotspot (Hoang et al., 2017). In this review, we focus on plant-specific HD-ZIP TFs, which play an essential role in plant responses to abiotic stress.

## The structure and classification of HD-ZIP proteins

**Figure 1:**
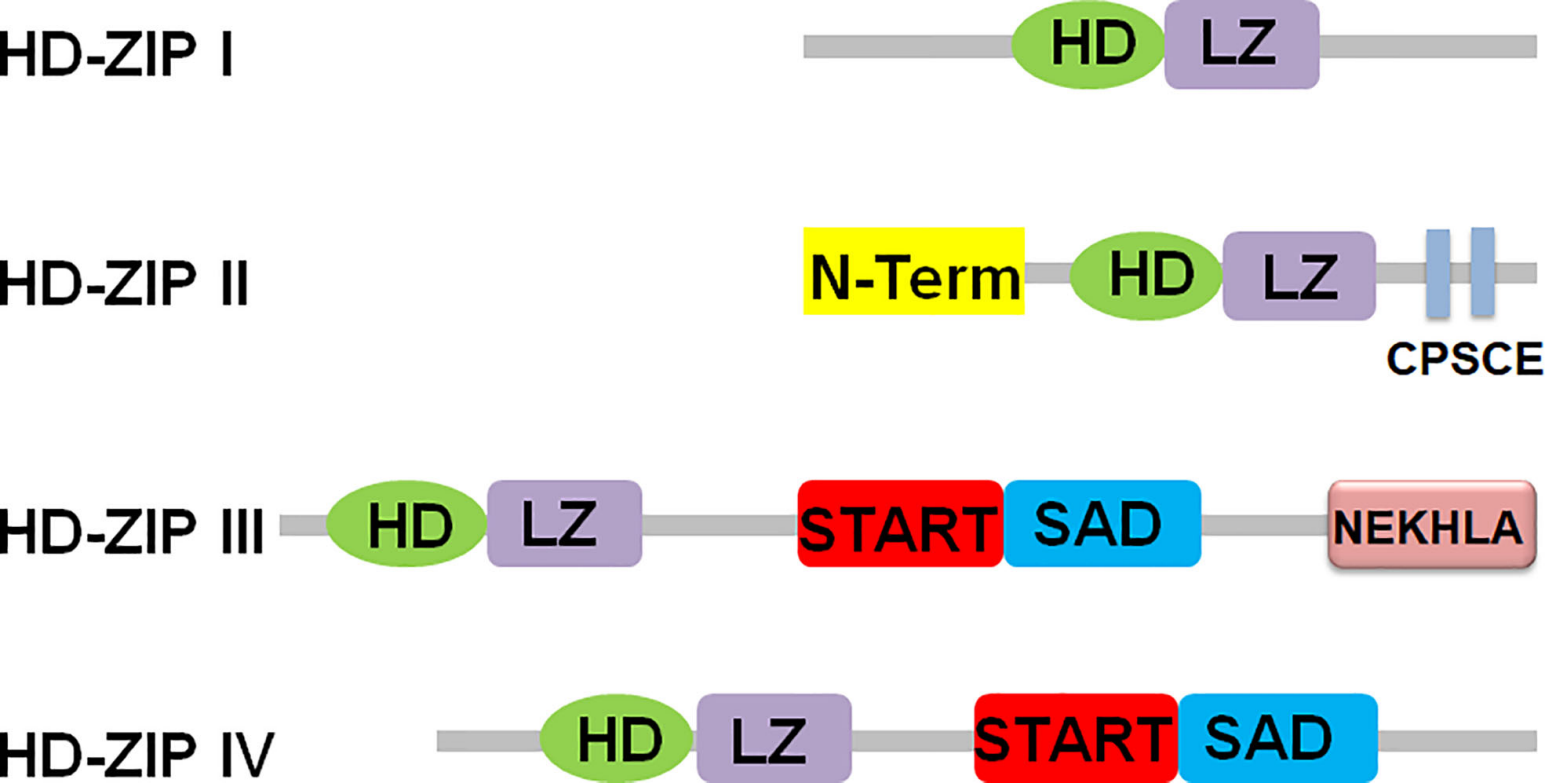
Schematic diagram of HD-ZIP family protein structure. HD, homeodomain; LZ, leucine-zipper; N-Term, N-term consensus sequence; START, steroidogenic acute regulatory proteinlipid transfer domain lipid transfer domain; MEKHLA, Met-Glu-Lys- His-Leu-Ala domain; SAD, START associated domain.

HD-ZIP transcription factors are members of the homeobox (HB) protein family. These TFs have a highly conserved homeodomain (HD) consisting of 61 amino acids, and a leucine zipper (LZ) element tightly linked to the carboxy terminus of HD. HD can specifically bind to DNA, and LZ mediates protein dimerization, which is important for DNA recognition (Xie et al., 2021). HD-ZIP proteins are divided into four subfamilies (I–IV) according to the specificity of DNA binding, sequence similarity, other conserved motifs, and differences in physiological functions (Sharif et al., 2021). The conserved domains and different motifs of four subfamilies of HD-ZIP proteins were shown in **Figure 1** (Sessa et al., 2018; Sharif et al., 2021). The HD-ZIP I protein contains a relatively poorly conserved LZ domain and a highly conserved HD domain. And its HD and LZ domains are located in the middle of the protein, which has the dual symmetric DNA sequence of CAAT(A/T)ATTG specific binding properties (Elhiti and Stasolla, 2009; Gong et al., 2019). Apart from the LZ and HD domains, members of the HD-ZIP I family differ from other family members in that they do not share domains and/or motifs. Many HD-ZIP I genes are involved in regulating signal transduction, regulating plant growth and development, and regulating plant tolerance to environmental stresses such as drought, salinity, cold, and heavy metal stress (Gong et al., 2019). Compared with HD-ZIP I, HD-ZIP II proteins have an N-terminal consensus sequence and a conserved CPSCE motif. The motif is named after five conserved amino acids (Cys, Pro, Ser, Cys and Glu). Moreover, HD-ZIP II protein has high homology with HD-ZIP I, and has specific binding properties to the double symmetrical DNA sequence of CAAT(C/G)ATTG (Elhiti and Stasolla, 2009; Gong et al., 2019). The HD domains of HD-ZIP I and II proteins have high sequence homology and both contain a highly conserved tryptophan-phenylalanine-glutamine-asparagine-arginine-arginine (WFQNRR) motif, the target DNA sequences recognized by the two differ by only one nucleotide in the middle. The specificity of this binding to a particular DNA element is primarily determined by the third helix, but the other two helices also contribute to this binding. By replacing Arg55 in helix 3 with Ala55, the protein cannot bind to DNA. Glu46 and Thr56 of helix 3 of HD-ZIP II protein are essential for this class of proteins to recognize C/G bases in DNA elements, while in HD-ZIP I protein, these two amino acid residues are replaced by Ala46 and Trp56, respectively (Ariel et al., 2007; Elhiti and Stasolla, 2009; Gong et al., 2019). The expression of HD-ZIP II protein is mainly regulated by photochemical conditions, and its conserved domain CPSCE motif is involved in regulating plant responses to light, shade and abiotic stresses (Park et al., 2013; Tognacca et al., 2021). Structurally, HD-ZIP III is very different from HD-ZIP I and II proteins, and has essentially the same domain as HD-ZIP IV protein. The HD-ZIP III and HD-ZIP IV subfamilies contain a steroidogenic acute regulatory protein-related lipid transfer (START) domain and a START-associated domain (SAD). The START domain responds to abscisic acid (ABA). However, the function of the SAD is unclear. HD-ZIP III and HD-ZIP IV proteins differ in that the C-terminus of HD-ZIP III protein contains the MEKHLA domain associated with various chemical and physical stimuli, whereas HD-ZIP IV protein does not contain this structure (Zhu et al., 2018). The HD-ZIP III protein can recognize the GTAAT(G/C)ATTAC sequence, while the HD-ZIP IV protein can interact with the TAAATG(C/T)A sequence (Elhiti and Stasolla, 2009; Gong et al., 2019).

Genes in the same subfamily usually tend to be conserved in function because their structures are relatively similar. Different subfamilies have different gene structures and encode diverse proteins, so their roles in plant growth and development are also different. The expression of HD-ZIP genes is subject to different regulatory mechanisms. For example, HD-ZIP I family genes play critical roles in regulating plant development, regulation of signaling networks, and signaling networks triggered by endogenous and external stimuli in response to various abiotic stresses (Gong et al., 2019). HD-ZIP II family genes are involved in plant organ development, photoprotection and hormone response, and their expression is regulated by light signals (Qiu et al., 2022). Genes of the HD-ZIP III subfamily are post-transcriptionally regulated by the microRNAs (miRNAs) miR165/166 (Emery et al., 2003; Carlsbecker et al., 2010; Fan et al., 2021). At least six distinct plant hormones interact to dynamically regulate the spatiotemporal expression of miR165/166 and HD-ZIP III subfamily proteins during Arabidopsis development (Sessa et al., 2018). It has been shown that the HD-ZIP III protein directly and positively regulated the expression of HD-ZIP II genes (Turchi et al., 2015). HD-ZIP IV subfamily genes play essential roles in epidermal cell differentiation and root development (Qiu et al., 2022).

## Subcellular localization of HD-ZIP proteins

A protein’s subcellular localization indicates its function. The majority identified HD-ZIP family members localize in the nucleus and directly regulate genes expression by binding to *cis*-elements in their target promoters (Zou et al., 2011). For example, the HD-ZIP I subfamily members AtHB12 in Arabidopsis (Shin et al., 2004), ZmHDZ1 in maize (*Zea mays*) (Wang et al., 2017), PtHB13 in trifoliate orange (*Citrus trifoliata*) (Ma et al., 2020), OsHOX12 and OsHOX14 in rice (*Oryza sativa*) (Shao et al., 2018), JcHDZ07 (Tang et al., 2019a) and JcHDZ16 (Tang et al., 2019b) in physic nut (*Jatropha curcas*), NtHD-ZIP I in tobacco (*Nicotiana tabacum*) (Li et al., 2019e), and CaATHB-12 in pepper (*Capsicum annuum*) (Zhang et al., 2020) are all nuclear-localized proteins. HD-ZIP II subfamily members HAT22/ABIG1 in Arabidopsis (Liu et al., 2016), CaHB1 in pepper (Oh et al., 2013), MaHDZII.4 and MaHDZII.7 in *Musa accuminata* (Yang et al., 2020), and the OCL1 in the HD-ZIP IV subfamily in maize (Depège-Fargeix et al., 2011) and ROC4 (Wei et al., 2016) in rice are also nuclear-localized proteins. Yeast one-hybrid screen identifies the HD-ZIP I transcription factor PtHB13 in citrus as an upstream regulator of FLOWERING LOCUS C (PtFLC). Citrus flowering is regulated by PtHB13 through binding to the *PtFLC* promoter (Ma et al., 2020). *OsHOX12* and *OsHOX14* belong to the HD-ZIP I family of genes that bind a specific DNA sequence AH1 CAAT (A/T) ATTG to activate its downstream reporter gene expression (Shao et al., 2018). PtHB13, OsHOX12 and OsHOX14 all function in the nucleus and directly regulate the expression of their downstream genes by binding to *cis*-acting elements on downstream target promoters. Under salt treatment, *JcHDZ07* negatively regulates transgenes by regulating the expression of several stress-responsive genes, such as *AtDREB1A*, *AtDREB2A*, *AtHKT1;1*, Δ1-Pyrroline-5-carboxylate synthetase 1 (*P5CS1*), *AtSOS1*, *AtSOS3*, *AtNHX1* and *AtAPX1* (Tang et al., 2019a). This indicates that JcHDZ07 protein makes plants more sensitive to salt stress by inhibiting the expression of salt-responsive genes. *HAT22/ABIG1* represses *HRE2* transcription by acting on a negative *cis*-regulatory element in the *HRE2* promoter in response to plant hypoxia and/or salt stress (Seok et al., 2022). These results indicates that HD-ZIPs localized in the nucleus mainly act as a transcription factor to regulate downstream target genes. In addition, although some individual HD-ZIP genes are localized and expressed in the nucleus, they interact with proteins in a certain way to exert their transcription factor functions. Transcriptional activation or repression by transcription factors is accompanied by changes in chromatin structure that enable transcription factors to gain access to their target promoters, a step that often requires multi-protein complexes capable of manipulating nucleosome structure. OUTER CELL LAYER1 (OCL1) is a nuclear-localized transcription factor with transcriptional activation activity that interacts with SWI3C1 resulting in chromatin opening and active transcription. SWI3C1 is a component of the SWI/SNF chromatin remodeling complex and is a C subfamily protein of plant SWI3 proteins (Depège-Fargeix et al., 2011).

There are also some HD-ZIP transcription factors that are distributed in both the cytoplasm and the nucleus, and its distribution is determined by its unique N-terminus. For example, the HD ZIP I protein MdHB1 in apple (*Malus × domestica*) (Jiang et al., 2017), HD ZIP II protein ATHB17 in Arabidopsis (Zhao et al., 2017) and HD ZIP IV protein CsGL2-LIKE in cucumber (*Cucumis sativus*) (Cai et al., 2020). MdHB1 can interact with WD40, MYB and bHLH in the cytoplasm to function as a homodimer and indirectly regulate gene expression. MdHB1 confines MdTTG1, MdMYB10 and MdbHLH3 to the cytoplasm and indirectly represses the transcription of *MdDFR* and *MdUFGT*. Once *MdHB1* is silenced, these transcription factors are released, which in turn activate *MdDFR* and *MdUFGT* expression, and promotes anthocyanin biosynthesis (Jiang et al., 2017). However, some HD-ZIP transcription factors expressed in the cytoplasm and nucleus, whose functions in the cytoplasm were not expressed, still function as transcription factors in the nucleus. For example, ATHB17 is a transcriptional repressor containing an EAR (ERF-associated repression of amphipathic)-like motif. *ATHB17* positively regulates plant tolerance to oxidative, drought and salt stress. *ATHB17* is a repressor of photosynthesis associated nuclear genes (*PhANGs*) and it has been reported to suppress transcription of 26 *PhANGs* under normal or salt stress conditions, among which *PSBO1*, *LHB1B1*, *LHB1B2*, *LHCA2*, and *FDA6* are directly regulated by *ATHB17* (Zhao et al., 2017). *ATHB17* directly or indirectly represses the transcription of many *PhANGs* through binding to its target promoters. It was also found that *ATHB17* can directly bind to the promoter of *ATSIG5* to activate its transcription, and then its protein translocates to the chloroplast as an important regulator of many chloroplast genes, such as *psbA*, *psbB*, *psbC*, *psbD*, and *psBT* (Zhao et al., 2017). ATSIG5 is a transcription factor whose expression can be induced by various stresses including high light, salinity, osmotic stress and low temperature (Nagashima et al., 2004). Studies have shown that CsGL2-LIKE interacts with the jasmonic acid (JA) ZIM domain protein CsJAZ1 to form the HD-ZIP IV-CsJAZ1 complex involved in cucumber male flower development (Cai et al., 2020). These HD-ZIP genes function in both the cytoplasm and nucleus. This can illustrate that, in the cytoplasm, HD-ZIP proteins form heterodimers or homodimers with other proteins to promote their transcriptional specificity.

## HD-ZIP proteins in plant growth and development

The HD-ZIP proteins of Arabidopsis are the most thoroughly understood (Turchi et al., 2013; Carabelli et al., 2018). Genome-wide identification and expression profiling have helped to identify and functionally characterize many other HD-ZIP proteins in different plants, such as potato (*Solanum tuberosum* L.) (Li et al., 2019d), cucumber (*Cucumis sativus* L.) (Sharif et al., 2020), tobacco (Li et al., 2019e), sesame (*Sesamum indicum* L.) (Wei et al., 2019), tea plant (*Camellia sinensis* L.) (Shen et al., 2019), Japanese apricot (*Prunus mume* L.) (Li et al., 2019c), physic nut, and foxtail millet (*Setaria italica* L.) (Chai et al., 2018). HD-ZIP proteins participate in regulating plant growth and development, such as fruit development, and maturity, anthocyanin accumulation, flowering, vascular development, epidermal cell development.

HD-ZIP proteins regulate fruit ripening by regulating cell wall degradation and ethylene biosynthesis. The HD-ZIP I genes *MaHDZI.19* and *MaHDZI.26*, and the HD-ZIP II genes *MaHDZII.4* and *MaHDZII.7* in banana (*Musa acuminata*) are significantly up-regulated during fruit ripening. These four MaHDZs specifically localize in the nucleus and activate several maturation-related genes, including *MaACO5*, which is related to ethylene biosynthesis, and *MaEXP2*, *MaEXPA10*, *MaPG4*, and *MaPL4*, which are related to cell wall degradation (Yang et al., 2020). *LcHB2*, a member of the litchi (*Litchi chinensis*) HD-ZIP I subfamily, regulates fruit drop by directly activating the cell wall degradation-related genes *LcCEL2* and *8*, while the HD-ZIP I gene *LcHB3* regulates fruit drop by promoting ethylene biosynthesis (Li et al., 2019a; Li et al., 2019b). The HD-ZIP II gene *PpHB.G7* interacts with the promoters of the ethylene biosynthesis genes *PpACS1* and *PpACO1* to promote peach (*Prunus persica*) maturation by promoting ethylene biosynthesis (Gu et al., 2019). Silencing of MdHB1 leads to the accumulation of anthocyanins in apple pulp. MdHB1 normally confines MdTTG1, MdMYB10 and MdbHLH3 proteins to the cytoplasm. But when *MdHB1* was silenced, these transcription factors were released, which in turn activated the expression of *MdDFR* and *MdUFGT*, and promoted anthocyanin biosynthesis, resulting in red-fleshed apple fruit (Jiang et al., 2017).

HD-ZIP proteins participate in induction of flowering and regulate flowering time. *HaHB10* in the sunflower (*Helianthus annuus* L.) is an HD-ZIP II family gene that responds to dark and light conditions and is involved in plant flowering induction. It mediates the vegetative-reproductive transition by activating specific flowering-related genes in response to salicylic acid (Rueda et al., 2005; Dezar et al., 2011). The HD-ZIP IV subfamily member *ROC4* promotes flowering in rice (Wei et al., 2016), while heterologous expression of the maize (*Zea mays*) HD-ZIP IV subfamily gene *OCL1* in rice delayed flowering time (Depège-Fargeix et al., 2011). *RICE FLOWERING LOCUS T 1* (*RFT1*), *Heading date 3a* (*Hd3a*), and *Early heading date 1* (*Ehd1*) promote flowering under long-day conditions in rice, and ROC4 indirectly inhibits the expression of these genes (Wei et al., 2016). HD-ZIP proteins are also involved in regulating the fertility of germ cells. The HD-ZIP IV subfamily member *CsGL2-LIKE* in cucumber is expressed in male flower buds and anthers (Cai et al., 2020). *CsGL2-LIKE* silencing represses the expression of the flower-forming gene, the flowering locus T (FT), resulting in partial male sterility, delayed flowering, and reduced pollen and seed vigour. CsGL2-LIKE also interacts with CsJAZ1 to regulate male flower development (Cai et al., 2020).

HD-ZIP proteins participate in vascular development. Five members of the Arabidopsis HD-ZIP III subfamily ATHB8, PHAVOLUTA/AtHB9 (PHV), PHABULOSA/AtHB14 (PHB), AtHB15/CORONA (CAN), and REVOL UTA/IFL1 (REV) are associated with vascular development (Baima et al., 1995; Prigge et al., 2005). Vascular defects are pronounced in *phb phv cna* triple mutant stems, and the loss-of-function mutation of *REV* leads to defective vascular development (Prigge et al., 2005). *ATHB8* overexpression lines promotes vascular cell differentiation in Arabidopsis (Baima et al., 2001).

Most HD-ZIP IV genes are specifically expressed in epidermal or sub-epidermal cells play important roles in the development and maintenance of the outer cell layer (Chew et al., 2013). Arabidopsis has 16 HD-ZIP IV subfamily members, including PROTODERMAL FACTOR2 (PDF2), ENHANCED DROUGHT TOLERANCE1/HOMEODOMAIN GLABROUS11 (AtEDT1/HDG11), HDG12, ARABIDOPSIS THALIANA MERISTEM LAYER1 (ATML1), and GLABRA2 (GL2). For example, GL2, a member of the Arabidopsis HD-ZIP IV subfamily, plays a critical role in the development of trichomes and root hairs. It acts as a negative regulator in the fate determination and initiation stage of root hairs (Song et al., 2011; Khosla et al., 2014). Kale lines overexpressing the HD-ZIP IV family gene *AtEDT1/HDG11* produced more root hairs and larger root structures, increasing their tolerance to drought and osmotic stress compared to wild type (Zhu et al., 2016).

HD-ZIP genes also regulate plant organ development. The overexpression and mutant studies of *ATHB13* and *ATHB23* genes showed that they are involved in the regulation of inflorescence stem, silique, ovule and seed development (Ribone et al., 2015). Arabidopsis *HomeoBox 1* (*AtHB1*) is mainly expressed in hypocotyls and roots and is up-regulated in short-day-grown seedlings (Capella et al., 2015). Compared with wild-type Arabidopsis, *AtHB1* knockdown mutants and overexpression lines exhibited shorter and longer hypocotyls, respectively. And *AtHB1* acts downstream of *PHYTOCHROME-INTERACTING FACTOR 1* (*PIF1*) to promote hypocotyl elongation (Capella et al., 2015).

## HD-ZIP proteins in plant abiotic stress

HD-ZIP family TFs are involved in the regulation of plant abiotic stress. In the model legume *Medicago truncatula* L., salt stress induces the expression of the HD-ZIP I transcription factor MtHB1 (Ariel et al., 2010). The researchers demonstrated through bioinformatics analysis that the *CsHDZ* gene in tea (*Camellia sinensis*) responds to ABA, high temperature, low temperature, high salinity and drought. The expression patterns of *CsHDZ* genes are related to the abiotic stress treatments they are subjected to. Expression of most selected *CsHDZ* genes was significantly increased under various stress treatments (Shen et al., 2019). The researchers identified 45 HD-ZIP genes in sesame (*Sesamum indicum* L.) seeds, named as *SiHDZ01-SiHDZ45*. Analysis of *SiHDZ* gene expression showed that these genes have different expression patterns in different tissues. More than half of *SiHDZ* genes were found to be differentially expressed under salt and drought stress, while HD-ZIP I and HD-ZIP II family genes play important roles in sesame response to stress (Wei et al., 2019). The HD-ZIP gene of potato (*Solanum tuberosum* L.) is involved in all stages of its growth and development. The study showed that 13 HD-ZIP genes of potato were expressed differently in different tissues at high temperature (37°C) and low temperature (4°C). Under 37°C high temperature treatment, the expression levels of *StHOX11* and *StHOX28* in leaves, *StHOX25* in stems, and *StHOX20*, *StHOX23* and *StHOX43* in roots were significantly up-regulated. Under low temperature conditions, the expression of *StHOX39* in stems and *StHOX1* and *StHOX20* in roots and leaves were significantly increased (Li et al., 2019d). Studies have shown that in *Craterostigma plantagineum*, the expression of all *CPHB* gene families is regulated by leaf and root dehydration. HD-ZIP I family members *CPHB-6* and *CPHB-7* accumulated in leaves at the initial stage of dehydration, but their expression decreased after prolonged dehydration. HD-ZIP I family members *CPHB-4/5* and HD-ZIP II family *CPHB-3* were down-regulated by dehydration in both leaves and roots. This suggests that HD-ZIP plays a role in regulating gene expression of desiccation tolerance in *Craterostigma plantagineum* (Deng et al., 2002).

## HD-ZIP proteins in drought tolerance

**Figure 2:**
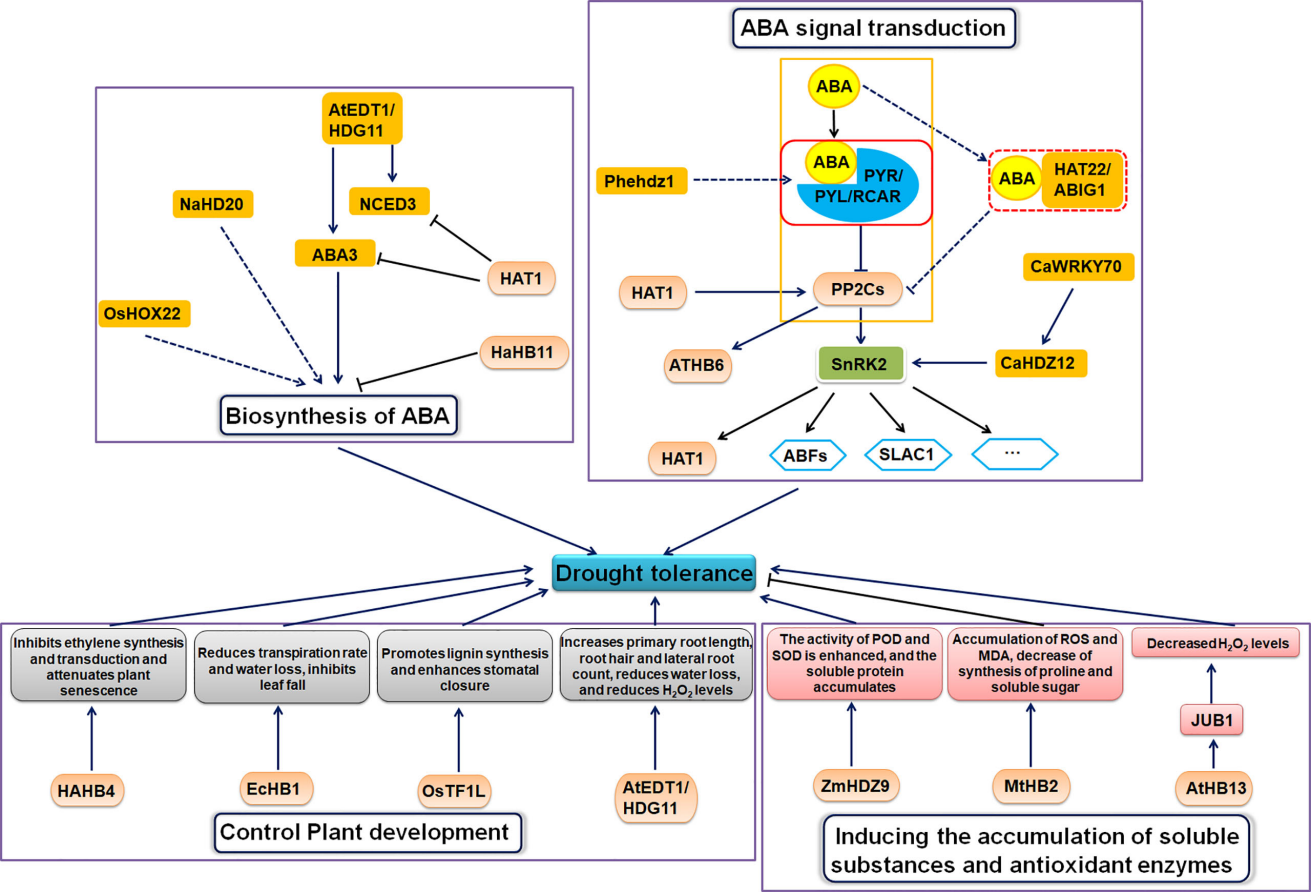
Molecular mechanism of HD-ZIP protein under drought stress.

Due to global climate change, drought stress has received increasing attention as one of the most harmful abiotic stresses to plants (Yao et al., 2020). Drought stress affects plant physiology, biochemistry, and metabolism, damaging cells, stunting growth, and reducing crop yield and quality (Gupta et al., 2020). HD-ZIP proteins facilitate plant adaptation to drought stress in multiple ways (**Figure 2**).

## HD-ZIP proteins regulate drought stress through the ABA signaling pathway

Some HD-ZIP transcription factors specifically bind ABA signaling or *cis*-elements in stress-responsive gene promoters to regulate drought response upon induction by exogenous ABA and drought stress (Mehmood et al., 2022).

### Roles in biosynthesis of ABA

ABA is a 15-carbon sesquiterpene generated by the cleavage of carotenoids in the 2-C-Methyl-D-erythritol 4-phosphate (MEP) pathway. Short-chain acyl-CoA dehydrogenase (SCAD), zeaxanthin cyclooxygenase (ZEP), and 9-cis-epoxycarotenoid dioxygenase (NCED) play key roles in ABA biosynthesis (Marin et al., 1996; Seo and Koshiba, 2002; Marusig and Tombesi, 2020).

Some HD-ZIP TFs alter plant tolerance to drought stress by altering ABA biosynthesis. Virus-induced gene silencing (VIGS) experiments show that the HD-ZIP I gene *NaHD20* promotes the expression of dehydration response genes *NaNCED1*, *NaOSM1* and *NaLTP1* and the accumulation of ABA in tobacco (*Nicotiana attenuata*) leaves under water stress (Ré et al., 2011). *OsHOX22* encodes a rice HD-ZIP I transcription factor that binds to the CAAT(G/C)ATTG element and acts as a transcriptional activator, requiring the HD and ZIP domains (Zhang et al., 2012). *OsHOX22-*overexpressing transgenic rice lines have increased sensitivity to ABA. Down-regulation of *OsHOX22* reduces ABA content and enhances drought tolerance in plants, whereas *OsHOX22* overexpression leads to increased ABA synthesis and accumulation and reduces drought tolerance (Zhang et al., 2012). The experimental results suggest that *OsHOX22* negatively regulates drought tolerance by increasing ABA biosynthesis. The HD-ZIP II gene homeodomain-leucine zipper protein 1 (*HAT1*) is involved in the regulation of drought tolerance in Arabidopsis (Tan et al., 2018). The *hat1* and *hat3* mutants have enhanced drought tolerance (Tan et al., 2018). HAT1 directly binds to the promoters of the ABA biosynthesis genes *ABA3* and *NCED3* to negatively regulate their expression, resulting in reduced ABA accumulation (Tan et al., 2018). The expression of the sunflower HD-ZIP I gene *HaHB11* was up-regulated under ABA and drought treatments, and heterologous overexpression of *HaHB11* enhanced drought tolerance in Arabidopsis and alfalfa (*Medicago sativa*) (Cabello et al., 2017). HaHB11 differentially regulates genes involved in ABA biosynthesis (*ABA1* and *ABA2*), ABA-independent signaling pathways (*COR15A* and *COR47*), and cytoprotection (*EM6* and *RD29B*) (Ding et al., 2009; Msanne et al., 2011; Cabello et al., 2017). Heterologously overexpressing the Arabidopsis HD-ZIP IV gene *AtEDT1*/*HDG11* enhances drought tolerance in Chinese kale (*Brassica oleracea* var. alboglabra) (Zhu et al., 2016). *NCED3* and *LOS5*/*ABA3*, which encode key ABA biosynthesis enzymes, were up-regulated in the transgenic kale line under non-stress and drought stress. Drought stress also up-regulates the stress marker gene *RD29A*, the proline synthesis gene *P5CS*, and the stress tolerance-related gene *LEA* in this transgenic line. Furthermore, the ABA/stress-related genes *NAC3*, *NAC5*, *SNAC1*, *DREB2*, *ABI3*, and *ABI5* were also significantly up-regulated in the *AtEDT1/HDG11*-overexpression lines (Zhu et al., 2016).

### Roles in ABA signal transduction

The ABA signaling pathway consists of RCAR/PYR/PYL receptor proteins, PP2C protein phosphatases, SnRK2 kinases (such as SnRK2.2, SnRK2.3, and SnRK2.6), and their target substrates (Umezawa et al., 2010; Guo et al., 2011; Yao et al., 2020). When ABA binds to and activates its receptor, the receptor forms a trimer complex with PP2Cs, thus inhibiting PP2C activity and abolishing its repression of SnRK2s (Yao et al., 2020). Active SnRK2s further activate the transcription factor ABFs/AREB1 [The basic leucine zippers (bZIPs) transcription factor], thereby initiating the expression of downstream genes.

Some other HD-ZIP transcription factors regulate drought tolerance in plants through the ABA signaling pathway. *ATHB6* is an HD-ZIP I family gene that negatively regulates ABA signaling and is a target of the protein phosphatase ABA Insensitive 1 (*ABI1*), acting downstream of *ABI1*. ABI1, a protein phosphatase 2C, is a key component in ABA signaling (Himmelbach et al., 2002). *HAT1* promotes the expression of *PP2Cs* and negatively regulates the ABA-mediated drought response and ABA signaling (Tan et al., 2018). HAT1 is a also substrate of the SnRK2.3 kinase, and phosphorylation by SnRK2.3 promoted HAT1 degradation (Tan et al., 2018). Drought stress induces ABA production, which inhibits PP2C activity, releasing SnRK2s to further activate downstream HAT1 by phosphorylation (Tan et al., 2018). CaHDZ12 is an HD-ZIP I transcription factor from chickpea (*Cicer arietinum*), and *CaHDZ12* is expressed in roots and stems under drought stress (Sen et al., 2017). *CaHDZ12* causes histone acetylation at chromatin to promote decondensation and subsequent gene transcription. *CaHDZ12* functions in ABA-dependent signaling and promotes osmolyte production, reduces intracellular ROS levels and induces expression of SnRK2 kinase and other stress-related genes in response to abiotic stress tolerance in chickpea (Sen et al., 2017). Further research found that, the WRKY transcription factor CaWRKY70 acts upstream of CaHDZ12 and negatively regulates its transcription by binding to the W-box(es) in the promoter region of *CaHDZ12* (Sen et al., 2017). The wheat (*Triticum aestivum*) HD-ZIP I gene *TaHDZ5-6A* positively regulates the drought stress response (Li et al., 2020). Under drought stress, transgenic Arabidopsis heterologously overexpressing *TaHDZ5-6A* has a lower water loss rate than wild-type, and stomatal closure and proline levels increased faster in transgenic plants than in wild type (Li et al., 2020). *DREB2A*, *RD29A*, *RD29B*, *RD26*, and *PP2CA* were up-regulated in the transgenic Arabidopsis compared with wild type Arabidopsis in the absence of stress, and were further up-regulated in the transgenic Arabidopsis under drought stress. This suggests that *TaHDZ5-6A* may regulate the transcription of these ABA signaling genes under drought conditions, thereby inducing rapid stomatal closure and reducing water loss (Li et al., 2020). HD-ZIP proteins regulate drought stress through ABA signal transduction and MAPKs signaling pathway. Overexpressing a moso bamboo (*Phyllostachys edulis*) HD-ZIP I gene, *Phehdz1*, in rice caused a significant increase in proline as well as a significant decrease in malondialdehyde (MDA) content relative to wild-type plants after drought treatment, and *Phehdz1*-overexpressing rice plants had enhanced drought tolerance (Gao et al., 2021). The *Phehdz1* promoter region contains four ABA-response elements (ABREs), and *Phehdz1* overexpression increased rice sensitivity to exogenous ABA; therefore, *Phehdz1* may enhance drought tolerance through ABA signaling (Gao et al., 2021). The expression of MAPK signaling pathway genes, such as *MPK2*, *MPK4*, *MPK6*, *WRKY33*, *CIPK25*, and *PR1*, changed significantly in *Phehdz1*-overexpressing rice under drought stress (Gao et al., 2021). These results suggest that *Phehdz1* may affect secondary metabolism *via* integrated ABA and MAPK signaling pathways to increase drought tolerance (Gao et al., 2021). After drought treatment, expression of the stress- and ABA-responsive genes *AP37*, *DREB1*, *ERF3*, *OsMYB3*, *OsNAC10*, and *OsWRKY47* was higher in transgenic lines compared to wild-type plants (Gao et al., 2021). Kyoto Encyclopedia of Genes and Genomes (KEGG) pathway enrichment analysis revealed that many differentially expressed genes (DEGs) were associated with metabolic pathways, such as phenylpropane biosynthesis and diterpene biosynthesis, suggesting that *Phehdz1* overexpression negatively impacts the response to drought stress, especially phenylpropane biosynthesis (Dong et al., 2020).

The expression of HD-ZIP II protein HAT22/ABA-INSENSITIVE GROWTH 1 (ABIG1) increased with ABA and drought treatment, and it shared a similar regulatory pattern with two other known ABA signaling genes, *PYL6* and *CIPK12*, suggesting that *HAT22*/*ABIG1* may be part of the ABA signaling pathway. Additionally, *HAT22*/*ABIG1* is required for ABA-mediated growth inhibition and leaf yellowing, suggesting that *HAT22*/*ABIG1* is involved in plant responses to drought stress (Liu et al., 2016).

## HD-ZIP proteins regulate drought tolerance by controlling plant development

Multiple aspects of plant morphology and growth process affect drought tolerance, including leaf area, stomatal density, vascular structure, root development and plant senescence.

The sunflower HD-ZIP I subfamily *HAHB4*, most similar to the Arabidopsis genes *ATHB7* and *ATHB12*, is positively regulated by drought, ethylene and ABA (Manavella et al., 2008). *HAHB4* is involved in a conserved mechanism associated with ethylene-mediated senescence that increases plant tolerance to drought stress. Because overexpressing *HAHB4* transgenic Arabidopsis exhibited strong tolerance to water stress and lower sensitivity to external ethylene, the overexpression lines entered the senescence pathway later. Studies have shown that the expression of *HAHB4* has inhibitory effects on ethylene synthesis-related genes (such as *ACO* and *SAM*) and ethylene signaling-related genes (such as *ERF2* and *ERF5*) (Manavella et al., 2006; Manavella et al., 2008).

The HD-ZIP II gene *EcHB1* positively regulates drought tolerance in *Eucalyptus*, which may be related to whole-tree transpiration changes. *EcHB1* overexpression inhibits defoliation in *Eucalyptus* under drought stress, thus promoting growth (Sasaki et al., 2019). In the *EcHB1-2*-overexpressing lines, stem diameter was reduced and the number of leaves per stem area increased, but there was minimal effect on stomatal conductance (g_s_) (Sasaki et al., 2019). Stomatal density and size influence g_s_ (Kiyomizu et al., 2019), so the small change in g_s_ probably occurred because stomatal density and size were not changed (Sasaki et al., 2019). Since the leaf area was significantly reduced (0.5-fold), the leaf number was slightly increased (1.3-fold), and the tree-base transpiration rate was decreased (0.6-fold), this reduced the overall transpiration rate and moisture loss, which may prevent defoliation (Sasaki et al., 2019). Reduced defoliation during drought stress may aid rapid recovery after rehydration. In addition, the vascular structure of the stem of the *EcHB1* overexpression line changed, the stem radial diameter decreased, and the cell wall thickness increased (Sasaki et al., 2019). These changes increase xylem cell stiffness, but decrease the water conductivity of stems and therefore also reduce water loss during drought stress (Lachenbruch and McCulloh, 2014; Santini et al., 2016). In conclusion, *EcHB1* overexpression enhanced drought tolerance by reducing leaf area (Sasaki et al., 2019).

HD-ZIP proteins improve drought tolerance by increasing lignin biosynthesis. Rice *OsTF1L* belongs to the HD-ZIP IV subfamily (Bang et al., 2019). Lignin biosynthesis genes, such as *PMEI*, were up-regulated in *OsTF1L*-overexpressing lines and down-regulated in *OsTF1L*-silenced lines. Additionally, genes involved in the drought response and stomatal movement, such as *ONAC022*, was also up-regulated in *OsTF1L*-overexpression plants (Bang et al., 2019). *PMEI* encodes an enzyme that inhibits pectin demethyl esterification, and when overexpressed, can improve drought tolerance (An et al., 2008). ONAC022 is a rice NAC transcription factor that regulates stomatal closure to improve plant drought tolerance. *OsTF1L* overexpression enhances stomatal closure and improves plant drought tolerance (Hong et al., 2016; Bang et al., 2019). Researchers have identified five direct target genes of *OsTF1L*. Among them, *Nodulin protein*, *DHHC4*, *poxN/PRX38*, and *CASPL5B1* are related to lignin synthesis, and the *AAA-type ATPase* is involved in drought tolerance. These five genes were up-regulated when *OsTF1L* was transiently expressed in rice protoplasts (Ranocha et al., 2010; Roppolo et al., 2011; Qi et al., 2013; Roppolo et al., 2014; Bang et al., 2019). In conclusion, *OsTF1L* enhances drought tolerance by regulating the expression of drought-responsive genes, stomatal movement and lignin biosynthesis (Bang et al., 2019).

Sunflower HaHB11 encodes the HD-ZIP I transcription factor. Heterologous overexpression of *HaHB11* resulted in a leaf-rolled phenotype and longer roots in Arabidopsis compared to wild type. These plants experience increased stem lignification, faster stomatal closure and less water loss under drought stress (Cabello et al., 2017). HD-ZIP IV gene *AtEDT1*/*HDG11* overexpression in kale increased primary root length, increased the number of root hairs and lateral roots, decreased water loss, and reduced H_2_O_2_ levels to enhance drought tolerance (Zhu et al., 2016). Arabidopsis *AtEDT1/HDG11* is a HD-ZIP IV family gene. In addition, studies have shown that overexpressing *AtHDG11* in wild-type Arabidopsis and tobacco have altered stomatal density and root development, as well as increased ABA content and improved photosynthesis, resulting in increased drought tolerance and biomass (Yu et al., 2008). Other studies have also shown that AtHDG11 improves drought tolerance in sweetpotato, tall fescue, rice, cotton and poplar due to a series of beneficial changes at the morphological and physiological level of transgenic plants, such as developed root systems and reduced stomatal density (Cao et al., 2009; Ruan et al., 2012; Yu et al., 2013; Yu et al., 2016).

## HD-ZIP proteins regulate drought tolerance by inducing the accumulation of soluble substances and antioxidant enzymes

In addition to decreasing water loss by changes in plant morphology, plants counter drought stress by accumulating soluble substances such as proline; these soluble substances improve water retention and protect cell membranes (Jiang et al., 2020; Zhang et al., 2021a). Plants also increase cellular antioxidant capacity to limit damage due to reactive oxygen species production.

Heterologous overexpression of the maize HD-ZIP I family gene *ZmHDZ9* in Arabidopsis leads to higher peroxidase (POD) and superoxide dismutase (SOD) activities and more soluble protein accumulation under drought stress (Qiu et al., 2022). Overexpression of the Arabidopsis HD-ZIP I gene *AtHB13* and heterologous overexpression of the sunflower homolog *HaHB1* in Arabidopsis enhanced drought tolerance (Hanson et al., 2001; Ebrahimian-Motlagh et al., 2017). *AtHB13* may improve drought tolerance by directly regulating the expression of *JUNGBRUNNEN1* (*JUB1*), which encodes a NAC transcription factor that regulates H_2_O_2_ levels (Ebrahimian-Motlagh et al., 2017). *MtHB2* is an alfalfa HD-ZIP I family gene, and Arabidopsis lines heterologously overexpressing *MtHB2* abolished the synthesis of proline and soluble sugars, increased the accumulation of ROS and MDA, resulting in reduced osmotic regulation and greater oxidative damage under stress than in the wild type (Song et al., 2012). Therefore, HD-ZIP proteins function in positively and negatively regulating plant responses to drought stress; understanding the downstream regulatory networks underlying these positive and negative effects has important implications for improving drought tolerance in crops without incurring yield penalties.

## HD-ZIP proteins in salinity stress responses

High salinity inhibits plant growth and reduces crop productivity by limiting plants’ ability to take up water and causing toxic ionic imbalances and ROS accumulation. HD-ZIP transcription factors regulate salt stress responses through multiple pathways (Wang et al., 2017; Tang et al., 2019a).

## HD-ZIP proteins participate in the salt stress response by regulating ion homeostasis, compatible solute concentrations, and ROS scavenging

Plant salt stress responses include activating ion channels to regulate ion homeostasis, accumulating solutes to mitigate osmotic effects, and (as with drought stress responses), activating antioxidant defenses to limit oxidative damage.

The apple HD-ZIP I gene *MdHB7-like* positively regulates the salt tolerance of apple. Under salt stress, the relative electrolyte leakage (REL) and MDA content of leaves of *MdHB7-like* overexpressing lines were reduced compared with wild type (Zhao et al., 2021). Furthermore, the transgenic apple had lower H_2_O_2_ and $O_{2}^{-}$ concentrations and higher SOD and POD activities; *SOS1*, *SOS2*, *SOS3*, and *NHX1* expression was higher; the proline and soluble sugar content was significantly increased; and the Na^+^/K^+^ ratio in roots, stems, and leaves was significantly lower in the *MdHB7-like* overexpression lines than in the wild type (Zhao et al., 2021). Under salt and drought stress, cotton lines heterologously overexpressing *AtHDG11* significantly reduced MDA levels and elevated SOD and CAT activities, thus protecting transgenic plants from oxidative damage by enhancing their ROS scavenging ability (Yu et al., 2016). Proline and soluble sugars are two important and effective compatible osmo-regulatory substances in higher plants and play an important role in the oxidative stress response of plants (Yu et al., 2016). Analysis of wild type and *JcHDZ07*-overexpressing lines under salt stress revealed that transgenic Arabidopsis overexpressing *JcHDZ07* exhibited lower proline content, lower CAT and SOD activities, and lower survival rates than wild-type. This indicated that overexpression of *JcHDZ07* enhanced the sensitivity of transgenic Arabidopsis to salinity stress (Tang et al., 2019a). *JcHDZ16* is a HD-ZIP I gene in physic nut. Compared with wild-type, rice lines heterologous overexpressing *JcHDZ16* had decreased proline content and activities of antioxidant enzymes such as catalase (CAT) and SOD and increased REL and MDA content, thus, the sensitivity of transgenic rice to salt stress was improved. In addition, the salt stress-responsive genes *OsHKT1;1*, *OsAPX2*, *OsDREB2A* and *OsP5CS* were expressed at lower levels in rice overexpressing *JcHDZ16* compared with wild type, suggesting that *JcHDZ16* negatively regulates ion balance and ROS scavenging under salt stress (Tang et al., 2019b). Overexpression of *PsnHDZ63*, the HD-ZIP I gene of *Populus simonii* × *P. nigra*, improves the salt tolerance of poplar (Guo et al., 2021). Under salt stress, *PsnHDZ63* positively regulates SOD and POD activities and reduces MDA content (Guo et al., 2021). Tomato HD-ZIP I subfamily gene *SlHB2*, whose expression is induced by ABA, 1-aminocyclopropane-1-carboxylic acid (ACC), methyl-jasmonic acid (MeJA), drought, high salt, low temperature and damage. *SlHB2* negatively regulates plant drought and high salinity tolerance. Compared with the wild type, under drought and salt stress, the chlorophyll and water contents in the leaves of the tomato seedlings of the *SlHB2-RNAi* line were higher, and the water loss rate and MDA content were lower, indicating that the *SlHB2-RNAi* line enhanced its stress resistance (Hu et al., 2017). The HD-ZIP II gene *CaHB1* of pepper enhances salt tolerance when heterologously expressed in transgenic tomato plants (Oh et al., 2013). The photosynthetic capacity was higher, and dehydrin transcripts were up-regulated 3- to 5-fold in the *CaHB1-*overexpressing lines compared to wild type plants (Oh et al., 2013).

## HD-ZIP transcription factors regulate salt tolerance through the ABA signaling pathway

ABA regulates plant responses to many abiotic stresses and its effects on drought and salt stress tolerance target similar types of damage induced by the two stresses, such as water balance and oxidate stress.

*GhHB1* is a cotton (*Gossypium hirsutum*) HD-ZIP I gene. Studies have shown that the expression of cotton *GhHB1* is up-regulated under ABA and NaCl treatments, suggesting that it may be involved in plant responses to salt stress and ABA signaling (Ni et al., 2008). Reduced salt tolerance in rice overexpressing the maize HD-ZIP I gene *ZmHDZ1* (Wang et al., 2017). In *ZmHDZ1*-overexpressing seedlings, exogenous ABA up-regulated *ZmHDZ1* expression and the transgenic seedlings had increased ABA sensitivity compared to WT (Wang et al., 2017). These results demonstrate that *ZmHDZ1* negatively regulates salt stress tolerance *via* an ABA-dependent signal transduction pathway (Wang et al., 2017). Salt stress and ABA treatment induced the expression of maize HD-ZIP I gene *ZmHDZ10*. Overexpression of *ZmHDZ10* in rice enhanced its resistance to drought and salt stress and increased its sensitivity to ABA. In addition, transgenic Arabidopsis overexpressing *ZmHDZ10* exhibited increased drought and salt tolerance and altered expression of stress/ABA-responsive genes, such as *RD29B*, *P5CS1*, *Responsive to Dehydration 22 (RD22)* and *ABI1*. These results suggest that the transcriptional regulator *ZmHDZ10* can enhance salt and drought tolerance in plants through an ABA-dependent signaling pathway (Zhao et al., 2014). *OsHOX22 and OsABI5* negatively regulate plant salt tolerance, while *OsLEA3* and *OsRAB16A* are downstream abiotic stress response genes that are often used as markers (Duan and Cai, 2012; Zhang et al., 2012; Li et al., 2016). Compared with wild-type, *ZmHDZ1*-overexpressing rice showed higher expression of *OsHOX22* and *OsABI5*, but lower expression of *OsRAB16A* and *OsLEA3* (Wang et al., 2017). *OsABI5* regulates ABA signaling, and its overexpression in rice increased sensitivity to salt stress (Zou et al., 2008). The proline content of alfalfa overexpressing the HD-ZIP I gene *MsHB7* was lower than that of wild-type alfalfa, which means that overexpression of *MsHB7* increases the sensitivity to salt stress (Li et al., 2022). The expression levels of the ABA-responsive genes *MsSnRK2.6* and *MsABI1* were lower in *MsHB7*-overexpressing lines after salt treatment compared to wild type (Li et al., 2022). *MsHB7* is induced *via* an ABA-dependent signaling pathway under salt stress. In addition to down-regulation of *MsSnRK2.6* and *MsABI1*, MsHB7 inhibits the antioxidant enzyme-encoding genes *MsCatalase4* and *MsPxdC* (Li et al., 2022). P5CS is the rate-limiting enzyme in proline biosynthesis, and *P5CS* expression is also dependent on ABA signaling under salt stress (Verslues and Bray, 2006). Transcript levels of *MsP5CS2* in *MsHB7*-overexpressing lines were lower compared to those in wild type, suggesting that *MsHB7* down-regulates *MsP5CS2* through an ABA-dependent signaling pathway (Li et al., 2022). The pepper HD-ZIP I genes *CaHDZ03* and *CaHDZ10* are salt-inducible expression genes (Zhang et al., 2021b). *CAHDZ03* contains two closely connected ABA action elements, indicating its potential role in the ABA signaling pathway (Zhang et al., 2021b).

Heterologously overexpressing the pepper HD-ZIP II gene *CaHB1* in tomato improved salt tolerance. Compared with wild-type, *CaHB1*-overexpressing tomato lines had higher photosynthetic capacity and also up-regulated the transcript level of dehydrin (Oh et al., 2013). *CaHB1* was not induced by ABA, implying that it may function in an ABA-independent manner.

Taken together, HD ZIP transcription factors play an essential role in plant salt resistance. It participates in salt stress response by enhancing ion homeostasis, osmotic stress and antioxidant capacity. HD-ZIP TFs can also be involved in the regulation of salt stress responses through ABA-mediated signaling. In addition, HD ZIP transcription factors were also found to be involved in plant responses to salt stress through a pathway independent of ABA signaling, such as regulating photosynthesis.

## HD-ZIP proteins in low-temperature stress

Cold stress limits plant development (Aslam et al., 2022) and leads to ROS and MDA production in cells. Low temperature stress affects cell membrane fluidity, consequently hindering photosynthesis, metabolism, and material transport (Janská et al., 2010; Ye et al., 2017). Expression profiling under abiotic stresses revealed that all 22 *SlHZI* genes in tomato respond to cold stress (Zhang et al., 2014b). Five HD-ZIP I genes were isolated from wheat, from *TaHDZipI-1* to *TaHDZipI-5* (Kovalchuk et al., 2016). Among them, when TaHDZipI-2 is overexpressed in barley (*Hordeum vulgare*), it affects cold tolerance by regulating cold-regulated expression of gene (COR gene) *HvTMC-AP3*, a putative chloroplastic amino acid selective channel protein (Kovalchuk et al., 2016). *TaHDZipI-5* is induced by cold and expressed at higher levels in flowers and early developing grains, indicating that *TaHDZipI-5* participates in cold tolerance during flowering (Yang et al., 2018). Using computer genome search, the researchers identified 37 HD-ZIP genes in *Dendrobium officinale* (*DoHDZs*), 34 *DoHDZs* were expressed under cold treatment, and 14 of them were significantly differentially expressed compared to control conditions, among them, 9 down-regulated genes and 5 up-regulated genes. The expression level of HD-ZIP I gene *DHDZ5* was significantly down-regulated under cold treatment, while HD-ZIP I gene *DoHDZ9* and HD-ZIP II gene *DoHDZ12* were significantly up-regulated, suggesting that they may be key candidates for cold stress response (Yang et al., 2022).

## HD-ZIP proteins participate in the low-temperature response by regulating cell membrane stability

The sunflower HD-ZIP I gene *HaHB1* and its Arabidopsis homolog *AtHB13* are associated with cold tolerance (Gong et al., 2019). Under freezing conditions, some antifreeze proteins (AFPs), such as PR2/PR4, accumulate in the apoplast of *HaHB1-*overexpressing Arabidopsis to improve low-temperature tolerance (Gong et al., 2019). In addition, transient transfection of sunflower and soybean (*Glycine max*) with *HaHB1* and *AtHB13* up-regulated their homologous target genes in these plants, indicating conservation of the cold-response pathway. This suggests that *HaHB1* and *AtHB13* are involved in cell membrane stability (Cabello et al., 2012; Cabello et al., 2017).

## HD-ZIP proteins regulate low-temperature tolerance through ROS scavenging

Pepper *CaATHB-12* encodes an HD-ZIP I transcription factor (Zhang et al., 2020). *CaATHB-12-*silenced fruit had high expression and activity of antioxidant enzymes, such as SOD and POD, suggesting its negative role in ROS scavenging. In addition, *CaATHB-12* regulates carotenoid biosynthesis; *CaATHB-12*-silenced pepper fruit had significantly lower carotenoid contents than wild-type fruit. Carotenoids participate in ROS scavenging (Lado et al., 2015). The expression levels of the stress-responsive genes *AtDREB2A*, *AtMYB44*, *AtRD29A* and *AtGPX3* were all lower in plants overexpressing *CaATHB-12* after cold stress than in wild type (Zhang et al., 2020). Transgenic Arabidopsis increased carotenoids, flavonoids, and phenolic compounds under cold stress by upregulating carotenoid biosynthesis genes (*CaPSY* and *CaLCYB*) (Zhang et al., 2020). Heterologous overexpression of *CaPSY* results in increased carotenoid accumulation in transgenic Arabidopsis (Rodriguez-Uribe et al., 2012; Zhang et al., 2020). *CaATHB-12* overexpression increases the carotenoid content under normal conditions; however, *CaATHB-12* overexpression impairs ROS scavenging under cold stress (Zhang et al., 2020).

## HD-ZIP proteins in regulating harmful metal stress

Aluminum (Al) stress due to excessive Al^3+^ limits plant growth and crop production in acidic soils. The earliest and most striking feature of Al poisoning is rapidly inhibited root elongation, which leads to reduced root size, restricted uptake of mineral nutrients and water, and ultimately reduced yield (Liu et al., 2014). The root transition zone (TZ) is located between the apical meristem of the root tip and the basal elongation zone, and this TZ senses Al toxicity (Rangel et al., 2007; Yang et al., 2014; Liu et al., 2020). Al mainly accumulates in the cell wall and changes cell wall properties, reduces its extensibility, and also prevents cell expansion in the root elongation zone (Horst et al., 2010). Cell wall pectin and hemicellulose bind Al (Blamey et al., 1990; Chang et al., 1999; Yang et al., 2011). Cell wall composition and structural modifications, especially those of pectin and xyloglucan oligosaccharides, affect the Al binding capacity and are important for Al stress tolerance (Lan et al., 2021).

Only a few TFs have been reported to be involved in Al tolerance. Two HD-ZIP I proteins, AtHB7 and AtHB12, were associated with Al tolerance *via* phenotypic analysis of overexpression lines and loss-of-function mutants (Söderman et al., 1996; Liu et al., 2020). AtHB7 and AtHB12 are closely related but their functions in Al resistance are diametrically opposed; *AtHB7* has a positive role and *AtHB12* has a negative role (Liu et al., 2020). AtHB7 and AtHB12 have opposite regulatory effects on Al stress tolerance, which is mediated by affecting Al accumulation in root cell walls. *ATHB12* increased the expression of the cell wall-related genes *DWARF4* (*DWF4*) and *EXPANSIN A10* (*EXPA10*) (Hur et al., 2015; Liu et al., 2020). Al accumulated in *athb7* roots, but did not accumulate in *athb12* roots. *AtHB7* and *ATHB12* form heterodimers and appear to inhibit each other in response to Al stress. The *athb7 athb12* double mutants are similar to the wild type in Al tolerance. Therefore, *AtHB7* and *AtHB12* may compete to regulate downstream Al-response genes (Liu et al., 2020). In addition, manganese (Mn) toxicity in acidic soils is also an important factor limiting crop yield. The researchers identified 63 and 87 Mn toxicity-response genes in *Citrus sinensis* and *Citrus grandis*, respectively. *Citrus grandis* is not resistant to Mn toxicity, while *Citrus sinensis* is resistant to Mn toxicity. Among them, manganese toxicity-induced root HD-ZIP I proteins (TDF #170-1 and 170-1k) showed a trend of up-regulation in both species (Zhou et al., 2016).

The heavy metal cadmium (Cd) is toxic to most organisms, including plants and humans (Ding et al., 2018). Cd is easily absorbed by plant roots, causing severe structural and functional changes, inhibiting seed germination and root growth, and Cd stress also increases ROS production (Farid et al., 2013; Ali et al., 2014; Rehman et al., 2022). HD-ZIP genes and their upstream microRNAs (miRNAs) are involved in Cd accumulation and tolerance. Under Cd stress, rice miR166 was down-regulated, while its target, the HD-ZIP III gene *OsHB4*, was up-regulated. Overexpressing miR166 in rice under Cd stress improved growth compared to the wild type, which was related to decreased Cd-induced oxidative damage and a significant increase in rice chlorophyll content. Overexpressing miR166 reduced Cd transport between roots and shoots (Ding et al., 2018). Accumulation of Cd in shoots and grains of rice mainly depends on the transport of xylem from roots to shoots (Uraguchi et al., 2009). This indicates that *OsHB4* is a target of miR166, and *OsHB4* regulates Cd translocation and tolerance in rice. Other genes (such as *OsHMA2* and *OsHMA3*) may also be involved in Cd absorption, storage, transport, and detoxification in rice.

## Mechanism of HD-ZIP proteins in regulating abiotic stress tolerance

Plants rapidly respond to adverse conditions *via* interconnected networks controlled by signaling cascades. Stress response involves signal perception and transduction and expression of stress-responsive genes (Baillo et al., 2019). Upon sensing a signal, two major signaling cascades in plants, the mitogen-activated protein kinase (MAPK) and calcium-dependent protein kinase (CDPK) pathways, are activated (Hernandez-Garcia and Finer, 2014; Erpen et al., 2018). ROS participate in signal transduction through MAPKs, and Ca^2+^ participates in signal transduction through the CDPK pathway, which then induce multiple pathways through the kinase cascade (Gilroy et al., 2016; Qi et al., 2018). These activated pathways regulate the expression of HD-ZIP genes, which then bind to cis-elements on target gene promoters to regulate downstream gene expression and subsequent biosynthesis of secondary metabolites (Baillo et al., 2019). Therefore, HD-ZIP transcription factors play a key role in responding to stress by directly regulating downstream target genes or interacting with other transcription factors (Kurusu et al., 2015; Gong et al., 2019; Chen and Yang, 2020; Qiu et al., 2022).

Interactions between the ABA pathway and other pathways (Yao et al., 2020), and interactions between ABA-dependent and ABA-independent pathways (Zhang et al., 2012; Cabello et al., 2017; Gong et al., 2019; Tang et al., 2019b) are involved in the stress response. ABA is rapidly synthesized under different stresses (Marcolino-Gomes et al., 2013). HD-ZIP proteins inhibit or promote ABA synthesis. HAT1 negatively regulates drought tolerance in Arabidopsis by directly binding to the promoters of the ABA biosynthesis genes *ABA3* and *NCED* (Tan et al., 2018). HaHB11 positively regulates drought tolerance by regulating the ABA biosynthesis genes *ABA1* and *ABA2* (Ding et al., 2009; Msanne et al., 2011; Cabello et al., 2017). *CaHDZ12* positively regulates drought tolerance by decreasing *CaPP2C* expression (Sen et al., 2017). *MsHB7* negatively regulates salt tolerance by inhibiting the expression of ABA-response genes *MsSnRK2.6* and *MsABI1* (Li et al., 2022)*. ZmHDZ1* negatively regulates salt tolerance in rice by increasing *Oshox22* and *OsABI5* expression (Wang et al., 2017). In addition to the ABA signaling pathway, HD-ZIP transcription factors such as *Phehdz1* may also affect secondary metabolism through an integration of ABA and MAPK signaling pathways to regulate drought tolerance (Gao et al., 2021).

Abiotic stresses stimulate ROS production in plant cells. HD-ZIP proteins also improve stress tolerance through promoting ROS scavenging. *ZmHDZ9-*overexpression in Arabidopsis resulted in higher SOD and POD activities under drought stress (Qiu et al., 2022). *AtHB13* directly regulates the expression of downstream *JUB1* to reduce cellular H_2_O_2_ levels under drought stress (Ebrahimian-Motlagh et al., 2017). *JcHDZ16* negatively regulates salt tolerance by decreasing *OsAPX2* expression and increasing *OsABI5* expression (ZHANG et al., 2014a; Tang et al., 2019b). HD-ZIP proteins protect macromolecules and subcellular structures by promoting the accumulation of osmotic substances, such as proline (Szabados and Savouré, 2010). For example, *MdHB7-like* increased proline content in plants under salt stress, while *AtEDT1*/*HDG11* increased proline content in plants under drought conditions (Zhu et al., 2016; Zhao et al., 2021).

HD-ZIP transcription factors also promote stress tolerance by regulating plant growth and development. *EcHB1* altered the anatomical structure of the stem vasculature, reduced the stem radial diameter, and increased cell wall thickness to improve drought stress (Sasaki et al., 2019). *OsTF1L* regulates lignin biosynthesis and enhances pore closure to improve drought (Bang et al., 2019). *CaHB1*-overexpression reduced chlorophyll loss under salt stress (Oh et al., 2013). HaHB1 enhanced cold tolerance by regulating cell membrane stability (Gong et al., 2019). *AtHB12* negatively regulates tolerance to Al by increasing the expression of cell wall-related genes (*EXPANDINA10* and *DWARF4*) (Liu et al., 2020). Pectin and hemicellulose in the cell wall bind Al to ameliorate Al toxicity (Blamey et al., 1990; Chang et al., 1999; Yang et al., 2011; Hur et al., 2015; Liu et al., 2020).

Different stresses may provoke similar responses. One HD-ZIP transcription factor may regulate multiple downstream genes, and one stress-response gene may be regulated by multiple HD-ZIP transcription factors. For example, *PheHDZ1* regulated the expression of numerous stress- and ABA-response genes, such as *AP37*, *DREB1*, *ERF3*, *OsMYB3*, *OsNAC10*, and *OsWRKY47*, to improve drought tolerance (Gao et al., 2021). *CaHDZ12* regulated the expression of tobacco ABA- and stress-response genes (*NtNCED1*, *NtRD26*, *NtDREB2A*, and *NtP5CS*) and antioxidant enzyme genes (*NtCatalase*, *NtSOD*, and *NtPOD*) to improve drought tolerance in transgenic tobacco (Sen et al., 2017). The expression of stress-response genes (*AtDREB2A*, *AtMYB44*, *AtRD29A*, and *ATGPX3*) were lower in *CaATHB-12-*overexpression transgenic Arabidopsis under cold stress (Zhang et al., 2020).

**Figure 3:**
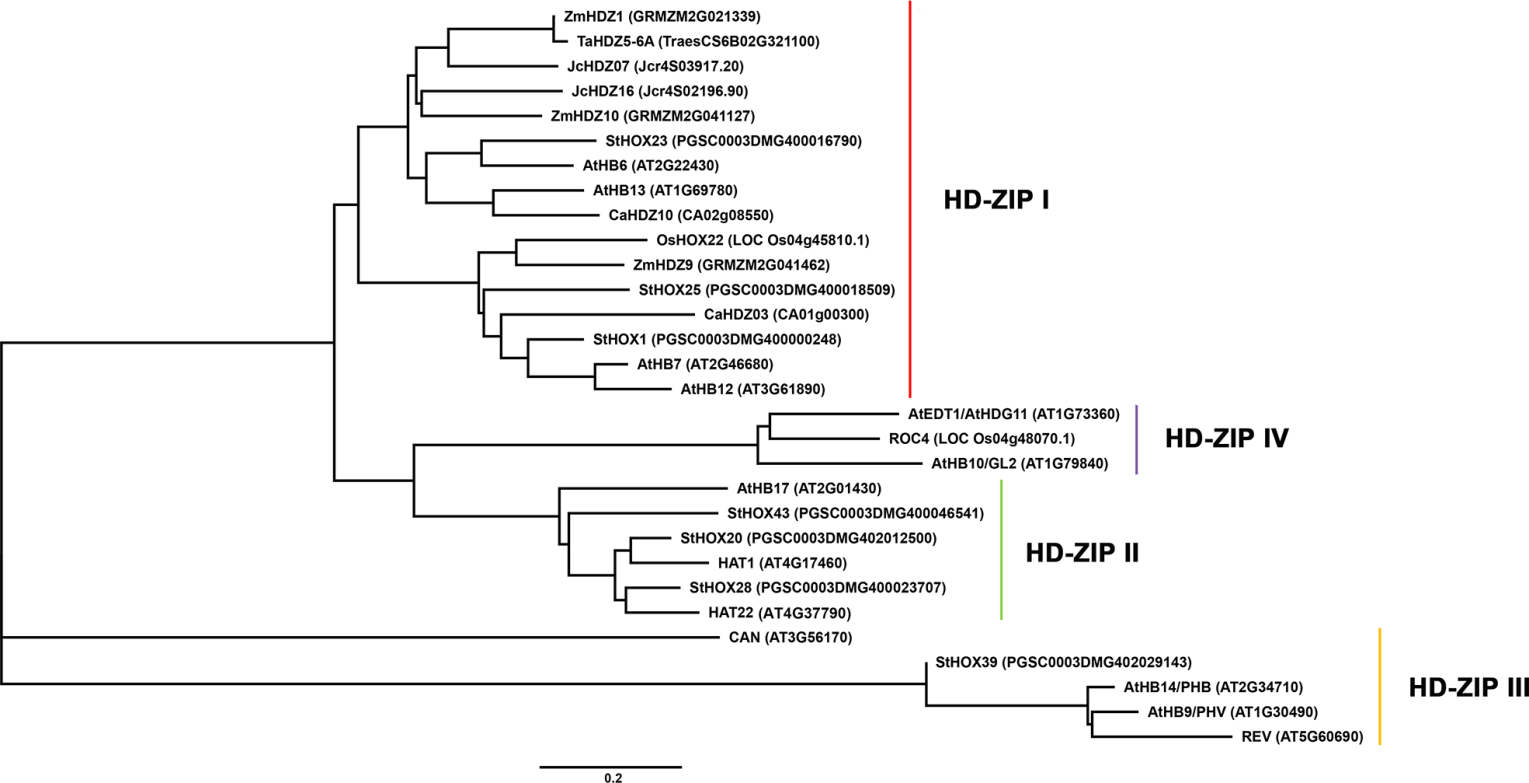
Phylogenetic tree analysis of HD-ZIP proteins in different species. The amino acid sequences of HD-ZIP proteins in different species were obtained from NCBI (https://www.ncbi.nlm.nih.gov/). MEGA 5.1 was used to construct a Neighbor-Joining phylogenetic tree with 1,000 bootstrap replicates.

Based on the above discussion on different subfamily proteins in stress tolerance, the conservative and different role of different subfamilies HD-ZIP proteins in resistance to abiotic stress in different species such as Arabidopsis, maize and rice was intuitively shown in a phylogenetic tree (**Figure 3**). Through phylogenetic tree analysis, it was found that HD-ZIP I proteins played an extremely important role in plant response to abiotic stress. Most HD-ZIP I subfamily proteins in plants such as model plant Arabidopsis and model crop maize respond to abiotic stresses such as salinity, drought, and osmosis through ABA-dependent or ABA-independent pathways (Hanson et al., 2001; Himmelbach et al., 2002; Wang et al., 2017). Followed by less HD-ZIP II subfamily proteins also play a role in plant responses to various abiotic stresses (Liu et al., 2016; Li et al., 2019d). Although HD-ZIP III and HD-ZIP IV subfamily genes are also characterized under stress, such as HD-ZIP III gene *StHOX39* is highly expressed under low temperature conditions (Li et al., 2019f). Overexpression of HD-ZIP IV gene *OsTF1L* and *AtEDT1/HDG11* both improved the drought tolerance of transgenic plants (Zhu et al., 2016; Bang et al., 2019). The HD-ZIP III and HD-ZIP IV genes are mainly involved in the regulation of plant development (Baima et al., 1995; Di Cristina et al., 1996; Masucci et al., 1996; Prigge et al., 2005; Khosla et al., 2014; Zhu et al., 2016).

**Figure 4:**
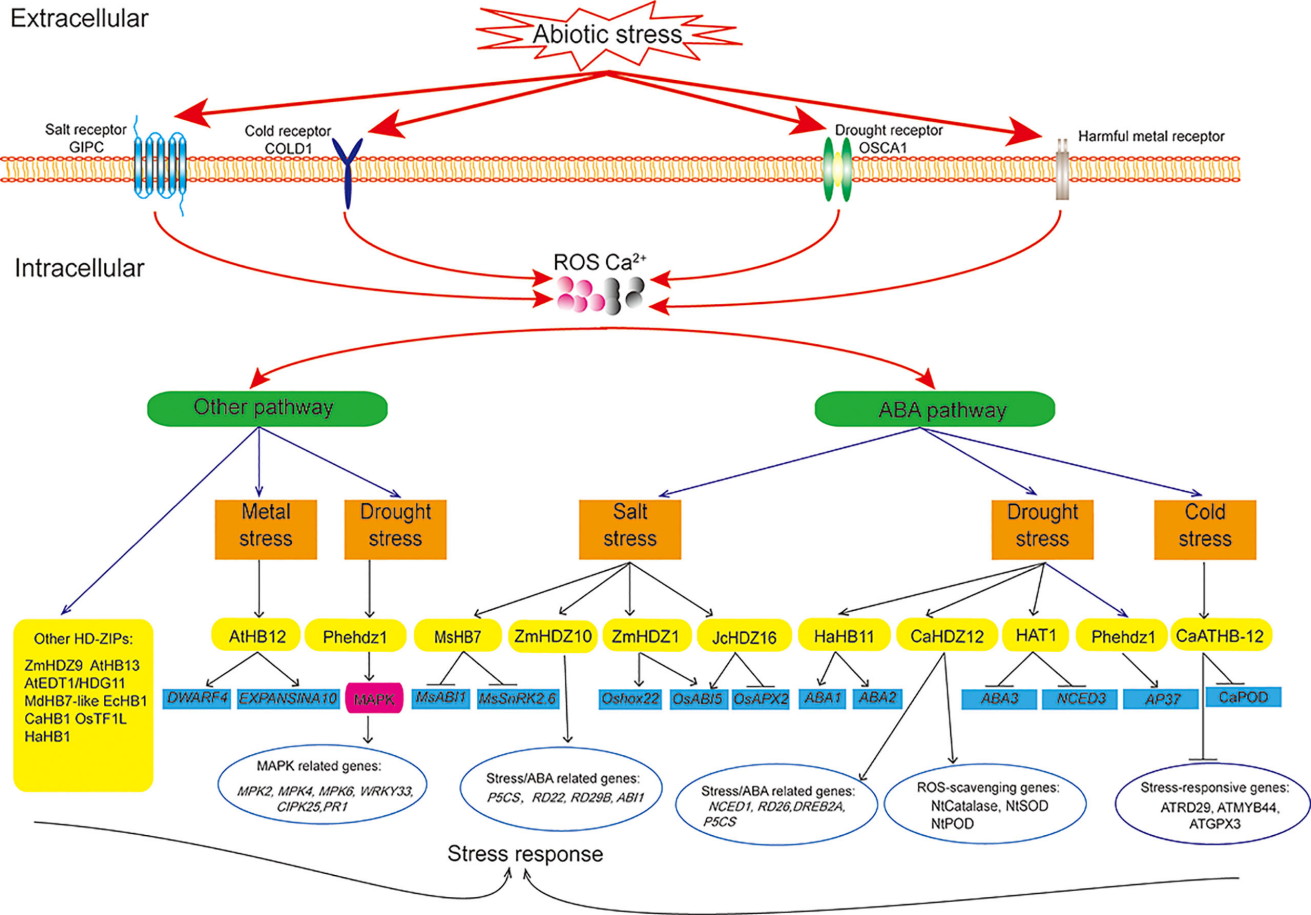
Molecular mechanism of HD-ZIP proteins under abiotic stress.

Through the above sorting and summary, the functions and regulation mechanisms of different types of HD-ZIP proteins involved in abiotic stress were detailed in **Supplementary Table 1**. And the regulatory network of HD-ZIP proteins in abiotic stress response was shown in **Figure 4**.

## Conclusions and future prospects

Considering that the world’s population is expected to reach 9 billion by 2050, improving crop tolerance to abiotic stresses is essential to meet the world’s projected food and energy needs. TFs have important roles in stress tolerance. Significant progress has been made in the role of transcription factor families in abiotic and biotic stress responses. In particular, HD-ZIP proteins play important roles in plant development and tolerance to abiotic stresses.

In recent years, great progress has been made in the study of plant HD-ZIP proteins. Since KNOTTED1 was first isolated from maize, researchers have now identified many HD-ZIP transcription factors from various plants. Although HD-ZIP family members have been extensively investigated in model plants and model crops, the molecular mechanisms in regulating abiotic stress are still insufficient. The upstream and downstream regulatory mechanisms of many HD-ZIP transcription factors remain unclear. Although genetic analysis shows that their stress response depends on the activation or inhibition of downstream target genes, they may also be regulated by their upstream genes, or work together through interacting proteins. However, scientific questions such as what is the upstream regulatory signal, how to form interacting proteins, how to regulate the expression of downstream genes, and what the regulatory network is all need to be solved. In addition, the response of HD-ZIP proteins to stress stimuli is extremely complex. Overexpressing a single HD-ZIP transcription factor may promote or inhibit the expression of multiple downstream genes. Different transcription factors may regulate the same gene, and a single transcription factor may respond to different abiotic stresses (Baillo et al., 2019). Identifying individual transcription factor genes at the molecular level is challenging. Multiple transcription factors can be studied by combining research methods and stresses to clarify the interactions between different transcription factors, rather than studying a single transcription factor and stress. Therefore, the development of biotechnologies such as CRISPR-Cas9, genome-wide association analysis, genome editing and single-cell sequencing will be very helpful to study the mechanism of HD-ZIP proteins in different species to improve plant abiotic stress tolerance. Chromatin immunoprecipitation followed by sequencing (ChIP-seq) and other biochemical technologies can be used to study the accurate binding sites of HD-ZIP transcription factors, and find the downstream functional genes regulated by them and the signal transduction pathways involved in plant stress tolerance.

Furthermore, it is important to study the mechanisms of stress adaptation in xerophytes and halophytes, especially HD-ZIP TFs in these plants. Due to the long-term growth and development of plants under adversity, stress tolerant plants have developed adaptability in their morphological anatomy, physiology and biochemistry, and can complete their life history under extreme adversity conditions. It is possible to study the stress tolerance function and mechanism of HD-ZIP transcription factors in xerophytes and halophytes. Isolating these key stress tolerance genes and transforming crops is of great significance for improving the stress tolerance of crops. Therefore, elucidating the molecular network regulation mechanism of HD-ZIP transcription factors in stress tolerant plants is also the focus of future scientific research, which can lay a foundation for the subsequent analysis of the biological functions of important plant transcription factors, and at the same time help to provide theoretical support and technical guidance for efficient screening of new crop varieties with resistance to abiotic stress.

Many plant developmental traits are also closely related to stress tolerance. HD-ZIP proteins are involved in the differentiation of plant trichomes and root hairs, and trichomes and root hairs participate in stress tolerance (Kang et al., 2014; Yang et al., 2015). Many HD-ZIP proteins participate in anthocyanins biosynthesis, and anthocyanins are involved in numerous abiotic stresses (Mbarki et al., 2018; Cirillo et al., 2021). In particular, the proteins of the HD-ZIP III and HD-ZIP IV subfamilies play a more important role in plant development. In the follow-up studies, the developmental phenotype and plant stress tolerance can be combined to better study the mechanism of these different HD-ZIP proteins in stress tolerance.

HD-ZIP proteins with better stress tolerance effect have been identified and characterized in more and more model plants and model crops. However, most of these studies were conducted in the laboratory. Under field conditions, crops are subject to a more complex and harsh environments. Therefore, there is an urgent need to test candidate these HD-ZIP proteins under field conditions to improve abiotic stress tolerance. Although analyzing plants overexpressing transcription factor genes under specific stresses is very informative, we need to assess the impact of these approaches on crop yield and productivity. Therefore, future research should not only explore whether stress-related transcription factor genes improve stress tolerance and growth, but also their effects on crop yield under natural field conditions.

## Authors contributions

YL and ZY wrote this manuscript. YZ, JG, LL and CW participated in the writing and modification of this manuscript. GH and BW conceptualized the idea. All authors read and approved the final manuscript.

## Funding

This work was supported by National Natural Science Research Foundation of China (project No. 32000209 and 32170301), Natural Science Research Foundation of Shandong Province (project No. ZR2020QC031) and China Postdoctoral Science Foundation (project No. 2020M672114).

## Conflict of interest

The authors declare that the research was conducted in the absence of any commercial or financial relationships that could be construed as a potential conflict of interest.

## Publisher’s note

All claims expressed in this article are solely those of the authors and do not necessarily represent those of their affiliated organizations, or those of the publisher, the editors and the reviewers. Any product that may be evaluated in this article, or claim that may be made by its manufacturer, is not guaranteed or endorsed by the publisher.

## Abbreviations

TF, transcription factor; HD-ZIP, homeodomain leucine zipper protein; HD, homologous domain; LZ, leucine zipper; DNA, Deoxyribonucleic acid; mRNA, Messenger RNA; ROS, reactive oxygen species; HB, homeobox; N-Term, N-term consensus sequence; START, steroidogenic acute regulatory proteinlipid transfer domain lipid transfer domain; MEKHLA, Met-Glu-Lys- His-Leu-Ala domain; SAD, START associated domain; PCR, polymerase chain reaction; ABA, Abscisic acid; MDA, malondialdehyde; ABRE, ABA response element; qRT-PCR, Quantitative Real-time PCR; WT, wild-type; DEGs, differentially expressed genes; KEGG, Kyoto Encyclopedia of Genes and Genomes; ChIP, Chromatin Immunoprecipitation; RNA, Ribonucleic acid; RNA-seq, RNA-sequencing; ChIP-seq, ChIP-sequencing; H_2_O_2,_ hydrogen peroxide; SOD, superoxide dismutase; POD, peroxidase; REL, relative electrolyte leakage; CAT, catalase; AFPs, antifreeze proteins; Al, Aluminum; TZ, transition zone; EZ, elongation zone; Cd Cadmium; miRNAs, microRNAs; Ca^2+^, calcium ions; MAPKs, mitogen-activated protein kinases; CDPK, calcium-dependent protein kinase; MEP, 2-C-Methyl-D-erythritol 4-phosphate; ZEP, Zeaxanthin cyclooxygenase; NCED, 9-cis-epoxycarotenoid dioxygenase; SCAD, short-chain acyl-CoA dehydrogenase.
