# Supplementary material for: The roles of HD-ZIP proteins in plant abiotic stress tolerance
Source: Front Plant Sci. 2022 Oct 12;13:1027071. doi: 10.3389/fpls.2022.1027071 (PMC9598875; doi:10.3389/fpls.2022.1027071)
Supplement: Supplementary file 1 [file Table_1.docx]

**Supplementary table 1** Function and regulation mechanism of HD-ZIP proteins in plant development and abiotic stress responses

| **Gene name** | **Plant species** | **Type** | **Function and regulatory mechanisms** | **Subcellular localization** | **References** |
| --- | --- | --- | --- | --- | --- |
| *StHOX1* | *Solanum tuberosum* L. | HD-ZIP I | Higher expression under low temperature stress |  | (Li et al., 2019) |
| *StHOX23* | *Solanum tuberosum* L. | HD-ZIP I | Higher expression under high temperature stress |  | (Li et al., 2019) |
| *StHOX25* | *Solanum tuberosum* L. | HD-ZIP I | Higher expression under high temperature stress |  | (Li et al., 2019) |
| *MtHB1* | *Medicago truncatula* L. | HD-ZIP I | Induced by salt stress |  | (Ariel et al., 2010) |
| *NaHD20* | *Nicotiana attenuata* | HD-ZIP I | Positively regulates plant drought stress response by promoting ABA biosynthesis |  | (Ré et al., 2011) |
| *GhHB1* | *Gossypium hirsutum* | HD-ZIP I | Response to salt stress and ABA signaling |  | (Ni et al., 2008) |
| *ATHB6* | *Arabidopsis thaliana* | HD-ZIP I | Negatively regulates plant drought stress responses through ABA signaling pathway | Nucleus | (Himmelbach et al., 2002) |
| *TaHDZ5-6A* | *Triticum aestivum* | HD-ZIP I | Positive regulation of plant drought stress response by regulating the transcription of ABA signaling genes |  | (Li et al., 2020) |
| *HAHB4* | *Helianthus annuus L.* | HD-ZIP I | Positive regulation of plant drought stress response by controlling plant development |  | (Manavella et al., 2006;Manavella et al., 2008) |
| *ZmHDZ9* | *Zea*  *mays* | HD-ZIP Ⅰ | Increase SOD and POD activity to improve drought tolerance | Nucleus | (Qiu et al., 2022) |
| *ZmHDZ10* | *Zea*  *mays* | HD-ZIP Ⅰ | Positively regulates plant drought and salt stress responses by the ABA signaling pathway | Nucleus | (Zhao et al., 2014) |
| *AtHB13* | *Arabidopsis thaliana* | HD-ZIP Ⅰ | Participates in low temperature response by regulating cell membrane stability;Positively regulates plant drought stress responses by regulating H_2_O_2_ levels | Nucleus | (Hanson et al., 2001;Ebrahimian-Motlagh et al., 2017;Gong et al., 2019) |
| *MdHB7-like* | *Malus domestica* | HD-ZIP I | Increase SOD and POD activity to improve salt tolerance | Nucleus | (Zhao et al., 2021) |
| *JcHDZ16* | *Jatropha curcas* | HD-ZIP Ⅰ | Negatively regulates plant salt stress response by negatively regulating ion homeostasis and ROS scavenging | Nucleus | (Tang et al., 2019b) |
| *JcHDZ07* | *Jatropha curcas* | HD-ZIP Ⅰ | Negatively regulates plant salt stress response by inhibiting the expression of salt-responsive genes | Nucleus | (Tang et al., 2019a) |
| *PsnHDZ63* | *Populus* | HD-ZIP Ⅰ | Increase SOD and POD activity to improve salt tolerance | Nucleus | (Guo et al., 2021) |
| *SlHB2* | *Lycopersicon esculentum* | HD-ZIP Ⅰ | Negatively regulates plant drought and salt stress responses by reducing MDA content and water loss rate |  | (Hu et al., 2017) |
| *ZmHDZ1* | *Zea*  *mays* | HD-ZIP Ⅰ | Negatively regulates plant salt stress responses through ABA signaling pathway | Nucleus | (Wang et al., 2017) |
| *MsHB7* | *Medicago sativa* | HD-ZIP Ⅰ | Negatively regulates plant salt stress through ABA dependent signaling pathway | Nucleus | (Li et al., 2022) |
| *CaHDZ03* | *Capsicum annuum* | HD-ZIP I | Positively regulates plant salt stress responses through ABA dependent signaling pathway |  | (Zhang et al., 2021) |
| *CaHDZ10* | *Capsicum annuum* | HD-ZIP I | Positively regulates plant salt stress responses |  | (Zhang et al., 2021) |
| *HaHB1* | *Helianthus annuus* | HD-ZIP I | Positively regulates plant cold stress responses by participating in cell membrane stability |  | (Cabello et al., 2012;Gong et al., 2019) |
| *TaHDZipI-2* | *Triticum aestivum* | HD-ZIP I | Positively regulates plant cold stress responses |  | (Kovalchuk et al., 2016;Yang et al., 2018) |
| *TaHDZipI-5* | *Triticum aestivum* | HD-ZIP I | Positively regulates plant cold stress responses |  | (Yang et al., 2018) |
| *CaATHB-12* | *Capsicum annuum* | HD-ZIP I | Negatively regulates plant cold stress responses by impairing the ROS scavenging pathway. | Nucleus | (Rodriguez-Uribe et al., 2012;Zhang et al., 2020) |
| *AtHB7* | *Arabidopsis thaliana* | HD-ZIP I | Affecting the accumulation of aluminum in root cell wall |  | (Söderman et al., 1996;Liu et al., 2020) |
| *AtHB12* | *Arabidopsis thaliana* | HD-ZIP I | Affecting the accumulation of aluminum in root cell wall |  | (Liu et al., 2020) |
| *PtHB13* | *Citrus* | HD-ZIP I | Inhibition of flowering of transgenic Arabidopsis | Nucleus | (Ma et al., 2020) |
| *MaHDZI.19* | *Musa accuminata* | HD-ZIP I | Role in fruit ripening | Nucleus | (Yang et al., 2020) |
| *MaHDZI.26* | *Musa accuminata* | HD-ZIP I | Role in fruit ripening | Nucleus | (Yang et al., 2020) |
| *LcHB2* | *Litchi chinensis* | HD-ZIP I | Positively regulates fruit abscission | Nucleus | (Ma et al., 2019) |
| *LcHB3* | *Litchi chinensis* | HD-ZIP I | Positively regulates fruit abscission | Nucleus | (Ma et al., 2019) |
| *MdHB1* | *Malus domestica* | HD-ZIP I | Negatively regulates anthocyanin biosynthesis | Cytoplasm and Nucleus | (Jiang et al., 2017) |
| *CPHB-4/5* | *Craterostigma plantagineum* | HD-ZIP I | Downregulated by dehydration in both leaves and roots |  | (Deng et al., 2002) |
| *CPHB-6/7* | *Craterostigma plantagineum* | HD-ZIP I | Higher expression during the early stages of dehydration |  | (Deng et al., 2002) |
| *StHOX20* | *Solanum tuberosum L.* | HD-ZIP II | Higher expression under high and low temperature stress |  | (Li et al., 2019) |
| *StHOX28* | *Solanum tuberosum* L. | HD-ZIP II | Higher expression under high temperature stress |  | (Li et al., 2019) |
| *StHOX43* | *Solanum tuberosum* L. | HD-ZIP II | Higher expression under high temperature stress |  | (Li et al., 2019) |
| *AtHB17* | *Arabidopsis thaliana* | HD-ZIP II | Positively regulates plant salt, drought and oxidative stress responses | Cytoplasm and nucleus | (Zhao et al., 2017) |
| *EcHB1* | *Eucalyptus camaldulensis* | HD-ZIP II | Inhibiting the defoliation under drought stress |  | (Sasaki et al., 2019) |
| *HAT22*/*ABIG1* | *Arabidopsis thaliana* | HD-ZIP II | Response to drought stress through the ABA signaling pathway | Nucleus | (Liu et al., 2016) |
| *HAT1* | *Arabidopsis thaliana* | HD-ZIP II | Negatively regulates plant drought stress response by participating in ABA biosynthesis and ABA signaling pathways |  | (Tan et al., 2018) |
| *CaHB1* | *Capsicum annuum* | HD-ZIP Ⅱ | Positively regulates plant photosynthetic capacity to improve salt tolerance | Nucleus | (Oh et al., 2013) |
| *HaHB10* | *Helianthus annus* | HD-ZIP II | Promote early flowering |  | (Rueda et al., 2005;Dezar et al., 2011) |
| *MaHDZII.4* | *Musa accuminata* | HD-ZIP II | Role in fruit ripening | Nucleus | (Yang et al., 2020) |
| *MaHDZII.7* | *Musa accuminata* | HD-ZIP II | Role in fruit ripening | Nucleus | (Yang et al., 2020) |
| *PpHB.G7* | *Prunus persica* | HD-ZIP II | Increase ethylene biosynthesis |  | (Gu et al., 2019) |
| *StHOX39* | *Solanum tuberosum L.* | HD-ZIP III | Higher expression under low temperature stress |  | (Li et al., 2019) |
| *REV* | *Arabidopsis thaliana* | HD-ZIP III | Role in vascular development |  | (Baima et al., 1995;Prigge et al., 2005) |
| *PHB* | *Arabidopsis thaliana* | HD-ZIP III | Role in vascular development |  | (Baima et al., 1995;Prigge et al., 2005) |
| *PHV* | *Arabidopsis thaliana* | HD-ZIP III | Role in vascular development |  | (Baima et al., 1995;Prigge et al., 2005) |
| *CAN* | *Arabidopsis thaliana* | HD-ZIP III | Role in vascular development |  | (Baima et al., 1995;Prigge et al., 2005) |
| *OsHB4* | *Oryza*  *sativa* | HD-ZIP III | Affecting the accumulation of chromium in cells |  | (Ding et al., 2018) |
| *OsTF1L* | *Oryza*  *sativa* | HD-ZIP Ⅳ | Regulating lignin biosynthesis to improve drought tolerance | Nucleus | (Bang et al., 2019) |
| *AtEDT1/HDG11* | *Arabidopsis thaliana* | HD-ZIP IV | Increase proline and SOD to improve drought tolerance |  | (Zhu et al., 2016) |
| *GL2* | *Arabidopsis thaliana* | HD-ZIP Ⅳ | Regulate the differentiation of trichomes and root hairs |  | (Di Cristina et al., 1996;Masucci et al., 1996;Khosla et al., 2014) |
| *HDG11* | *Arabidopsis thaliana* | HD-ZIP Ⅳ | Negatively regulates trichome branching and root hair development |  | (Khosla et al., 2014;Zhu et al., 2016) |
| *ROC4* | *Oryza*  *sativa* | HD-ZIP IV | Promote flowering time | Nucleus | (Wei et al., 2016) |
| *OCL1* | *Zea*  *mays* | HD-ZIP IV | Delayed flowering time | Nucleus | (Depege-Fargeix et al., 2011) |
| *CsGL2-LIKE* | *Cucumis sativus* | HD-ZIP IV | Regulating male flower development | cytosol and nucleus | (Cai et al., 2020) |

**References**

Ariel, F., Diet, A., Verdenaud, M., Gruber, V., Frugier, F., Chan, R., and Crespi, M. (2010). Environmental regulation of lateral root emergence in Medicago truncatula requires the HD-Zip I transcription factor HB1. *Plant Cell* 22**,** 2171-2183.

Baima, S., Nobili, F., Sessa, G., Lucchetti, S., Ruberti, I., and Morelli, G. (1995). The expression of the Athb-8 homeobox gene is restricted to provascular cells in Arabidopsis thaliana. *Development* 121**,** 4171-4182.

Bang, S.W., Lee, D.K., Jung, H., Chung, P.J., Kim, Y.S., Choi, Y.D., Suh, J.W., and Kim, J.K. (2019). Overexpression of OsTF1L, a rice HD-Zip transcription factor, promotes lignin biosynthesis and stomatal closure that improves drought tolerance. *Plant Biotechnol J* 17**,** 118-131.

Cabello, J.V., Arce, A.L., and Chan, R.L. (2012). The homologous HD-Zip I transcription factors HaHB1 and AtHB13 confer cold tolerance via the induction of pathogenesis-related and glucanase proteins. *Plant J* 69**,** 141-153.

Cai, Y., Bartholomew, E.S., Dong, M., Zhai, X., Yin, S., Zhang, Y., Feng, Z., Wu, L., Liu, W., and Shan, N. (2020). The HD-ZIP IV transcription factor GL2-LIKE regulates male flowering time and fertility in cucumber. *Journal of experimental botany* 71**,** 5425-5437.

Deng, X., Phillips, J., Meijer, A.H., Salamini, F., and Bartels, D. (2002). Characterization of five novel dehydration-responsive homeodomain leucine zipper genes from the resurrection plant Craterostigma plantagineum. *Plant Mol Biol* 49**,** 601-610.

Depege-Fargeix, N., Javelle, M., Chambrier, P., Frangne, N., Gerentes, D., Perez, P., Rogowsky, P.M., and Vernoud, V. (2011). Functional characterization of the HD-ZIP IV transcription factor OCL1 from maize. *Journal of experimental botany* 62**,** 293-305.

Dezar, C.A., Giacomelli, J.I., Manavella, P.A., Ré, D.A., Alves-Ferreira, M., Baldwin, I.T., Bonaventure, G., and Chan, R.L. (2011). HAHB10, a sunflower HD-Zip II transcription factor, participates in the induction of flowering and in the control of phytohormone-mediated responses to biotic stress. *Journal of experimental botany* 62**,** 1061-1076.

Di Cristina, M., Sessa, G., Dolan, L., Linstead, P., Baima, S., Ruberti, I., and Morelli, G. (1996). The Arabidopsis Athb‐10 (GLABRA2) is an HD‐Zip protein required for regulation of root hair development. *The Plant Journal* 10**,** 393-402.

Ding, Y., Gong, S., Wang, Y., Wang, F., Bao, H., Sun, J., Cai, C., Yi, K., Chen, Z., and Zhu, C. (2018). MicroRNA166 modulates cadmium tolerance and accumulation in rice. *Plant Physiology* 177**,** 1691-1703.

Ebrahimian-Motlagh, S., Ribone, P.A., Thirumalaikumar, V.P., Allu, A.D., Chan, R.L., Mueller-Roeber, B., and Balazadeh, S. (2017). JUNGBRUNNEN1 Confers Drought Tolerance Downstream of the HD-Zip I Transcription Factor AtHB13. *Front Plant Sci* 8**,** 2118.

Gong, S., Ding, Y., Hu, S., Ding, L., Chen, Z., and Zhu, C. (2019). The role of HD‐Zip class I transcription factors in plant response to abiotic stresses. *Physiologia plantarum* 167**,** 516-525.

Gu, C., Guo, Z.-H., Cheng, H.-Y., Zhou, Y.-H., Qi, K.-J., Wang, G.-M., and Zhang, S.-L. (2019). A HD-ZIP II HOMEBOX transcription factor, PpHB. G7, mediates ethylene biosynthesis during fruit ripening in peach. *Plant Science* 278**,** 12-19.

Guo, Q., Jiang, J., Yao, W., Li, L., Zhao, K., Cheng, Z., Han, L., Wei, R., Zhou, B., and Jiang, T. (2021). Genome-wide analysis of poplar HD-Zip family and over-expression of PsnHDZ63 confers salt tolerance in transgenic Populus simonii× P. nigra. *Plant Science* 311**,** 111021.

Hanson, J., Johannesson, H., and Engström, P. (2001). Sugar-dependent alterations in cotyledon and leaf development in transgenic plants expressing the HDZhdip gene ATHB13. *Plant molecular biology* 45**,** 247-262.

Himmelbach, A., Hoffmann, T., Leube, M., Höhener, B., and Grill, E. (2002). Homeodomain protein ATHB6 is a target of the protein phosphatase ABI1 and regulates hormone responses in Arabidopsis. *Embo j* 21**,** 3029-3038.

Hu, J.T., Chen, G.P., Yin, W.C., Cui, B.L., Yu, X.H., Lu, Y., and Hu, Z.L. (2017). Silencing of SlHB2 Improves Drought, Salt Stress Tolerance, and Induces Stress-Related Gene Expression in Tomato. *Journal of Plant Growth Regulation* 36**,** 578-589.

Jiang, Y., Liu, C., Yan, D., Wen, X., Liu, Y., Wang, H., Dai, J., Zhang, Y., Liu, Y., and Zhou, B. (2017). MdHB1 down-regulation activates anthocyanin biosynthesis in the white-fleshed apple cultivar ‘Granny Smith’. *Journal of Experimental Botany* 68**,** 1055-1069.

Khosla, A., Paper, J.M., Boehler, A.P., Bradley, A.M., Neumann, T.R., and Schrick, K. (2014). HD-Zip proteins GL2 and HDG11 have redundant functions in Arabidopsis trichomes, and GL2 activates a positive feedback loop via MYB23. *The Plant Cell* 26**,** 2184-2200.

Kovalchuk, N., Chew, W., Sornaraj, P., Borisjuk, N., Yang, N., Singh, R., Bazanova, N., Shavrukov, Y., Guendel, A., and Munz, E. (2016). The homeodomain transcription factor Ta HDZ ipI‐2 from wheat regulates frost tolerance, flowering time and spike development in transgenic barley. *New Phytologist* 211**,** 671-687.

Li, S., Chen, N., Li, F., Mei, F., Wang, Z., Cheng, X., Kang, Z., and Mao, H. (2020). Characterization of wheat homeodomain-leucine zipper family genes and functional analysis of TaHDZ5-6A in drought tolerance in transgenic Arabidopsis. *BMC plant biology* 20**,** 1-23.

Li, W., Dong, J., Cao, M., Gao, X., Wang, D., Liu, B., and Chen, Q. (2019). Genome-wide identification and characterization of HD-ZIP genes in potato. *Gene* 697**,** 103-117.

Li, X., Hou, Y., Li, M., Zhang, F., Yi, F., Kang, J., Yang, Q., and Long, R. (2022). Overexpression of an ABA-inducible homeodomain-leucine zipper I gene MsHB7 confers salt stress sensitivity to alfalfa. *Industrial Crops and Products* 177**,** 114463.

Liu, T., Longhurst, A.D., Talavera-Rauh, F., Hokin, S.A., and Barton, M.K. (2016). The Arabidopsis transcription factor ABIG1 relays ABA signaled growth inhibition and drought induced senescence. *Elife* 5, e13768.

Liu, Y., Xu, J., Guo, S., Yuan, X., Zhao, S., Tian, H., Dai, S., Kong, X., and Ding, Z. (2020). AtHB7/12 regulate root growth in response to aluminum stress. *International journal of molecular sciences* 21**,** 4080.

Ma, X., Li, C., Huang, X., Wang, H., Wu, H., Zhao, M., and Li, J. (2019). Involvement of HD-ZIP I transcription factors LcHB2 and LcHB3 in fruitlet abscission by promoting transcription of genes related to the biosynthesis of ethylene and ABA in litchi. *Tree physiology* 39**,** 1600-1613.

Ma, Y.-J., Li, P.-T., Sun, L.-M., Zhou, H., Zeng, R.-F., Ai, X.-Y., Zhang, J.-Z., and Hu, C.-G. (2020). HD-ZIP I transcription factor (pthb13) negatively regulates citrus flowering through binding to FLOWERING LOCUS C Promoter. *Plants* 9**,** 114.

Manavella, P.A., Arce, A.L., Dezar, C.A., Bitton, F., Renou, J.P., Crespi, M., and Chan, R.L. (2006). Cross-talk between ethylene and drought signalling pathways is mediated by the sunflower Hahb-4 transcription factor. *Plant J* 48**,** 125-137.

Manavella, P.A., Dezar, C.A., Ariel, F.D., Drincovich, M.F., and Chan, R.L. (2008). The sunflower HD-Zip transcription factor HAHB4 is up-regulated in darkness, reducing the transcription of photosynthesis-related genes. *J Exp Bot* 59**,** 3143-3155.

Masucci, J.D., Rerie, W.G., Foreman, D.R., Zhang, M., Galway, M.E., Marks, M.D., and Schiefelbein, J.W. (1996). The homeobox gene GLABRA2 is required for position-dependent cell differentiation in the root epidermis of Arabidopsis thaliana. *Development* 122**,** 1253-1260.

Ni, Y., Wang, X., Li, D., Wu, Y., Xu, W., and Li, X. (2008). Novel cotton homeobox gene and its expression profiling in root development and in response to stresses and phytohormones. *Acta Biochim Biophys Sin (Shanghai)* 40**,** 78-84.

Oh, S.-K., Yoon, J., Choi, G.J., Jang, H.A., Kwon, S.-Y., and Choi, D. (2013). Capsicum annuum homeobox 1 (CaHB1) is a nuclear factor that has roles in plant development, salt tolerance, and pathogen defense. *Biochemical and Biophysical Research Communications* 442**,** 116-121.

Prigge, M.J., Otsuga, D., Alonso, J.M., Ecker, J.R., Drews, G.N., and Clark, S.E. (2005). Class III homeodomain-leucine zipper gene family members have overlapping, antagonistic, and distinct roles in Arabidopsis development. *The Plant Cell* 17**,** 61-76.

Qiu, X., Wang, G., Abou-Elwafa, S.F., Fu, J., Liu, Z., Zhang, P., Xie, X., Ku, L., Ma, Y., and Guan, X. (2022). Genome-wide identification of HD-ZIP transcription factors in maize and their regulatory roles in promoting drought tolerance. *Physiology and Molecular Biology of Plants***,** 28, 425–437.

Ré, D.A., Dezar, C.A., Chan, R.L., Baldwin, I.T., and Bonaventure, G. (2011). Nicotiana attenuata NaHD20 plays a role in leaf ABA accumulation during water stress, benzylacetone emission from flowers, and the timing of bolting and flower transitions. *J Exp Bot* 62**,** 155-166.

Rodriguez-Uribe, L., Guzman, I., Rajapakse, W., Richins, R.D., and O’connell, M.A. (2012). Carotenoid accumulation in orange-pigmented Capsicum annuum fruit, regulated at multiple levels. *Journal of Experimental Botany* 63**,** 517-526.

Rueda, E.C., Dezar, C.A., Gonzalez, D.H., and Chan, R.L. (2005). Hahb-10, a sunflower homeobox-leucine zipper gene, is regulated by light quality and quantity, and promotes early flowering when expressed in Arabidopsis. *Plant and cell physiology* 46**,** 1954-1963.

Sasaki, K., Ida, Y., Kitajima, S., Kawazu, T., Hibino, T., and Hanba, Y.T. (2019). Overexpressing the HD-Zip class II transcription factor EcHB1 from Eucalyptus camaldulensis increased the leaf photosynthesis and drought tolerance of Eucalyptus. *Sci Rep* 9**,** 14121.

Söderman, E., Mattsson, J., and Engström, P. (1996). The Arabidopsis homeobox gene ATHB‐7 is induced by water deficit and by abscisic acid. *The Plant Journal* 10**,** 375-381.

Tan, W., Zhang, D., Zhou, H., Zheng, T., Yin, Y., and Lin, H. (2018). Transcription factor HAT1 is a substrate of SnRK2. 3 kinase and negatively regulates ABA synthesis and signaling in Arabidopsis responding to drought. *PLoS Genetics* 14**,** e1007336.

Tang, Y., Bao, X., Wang, S., Liu, Y., Tan, J., Yang, M., Zhang, M., Dai, R., and Yu, X. (2019a). A Physic Nut Stress-Responsive HD-Zip Transcription Factor, JcHDZ07, Confers Enhanced Sensitivity to Salinity Stress in Transgenic Arabidopsis. *Front Plant Sci* 10**,** 942.

Tang, Y., Wang, J., Bao, X., Liang, M., Lou, H., Zhao, J., Sun, M., Liang, J., Jin, L., Li, G., Qiu, Y., and Liu, K. (2019b). Genome-wide identification and expression profile of HD-ZIP genes in physic nut and functional analysis of the JcHDZ16 gene in transgenic rice. *BMC Plant Biol* 19**,** 298.

Wang, Q., Zha, K., Chai, W., Wang, Y., Liu, B., Jiang, H., Cheng, B., and Zhao, Y. (2017). Functional analysis of the HD-Zip I gene ZmHDZ1 in ABA-mediated salt tolerance in rice. *Journal of Plant Biology* 60**,** 207-214.

Wei, J., Choi, H., Jin, P., Wu, Y., Yoon, J., Lee, Y.-S., Quan, T., and An, G. (2016). GL2-type homeobox gene Roc4 in rice promotes flowering time preferentially under long days by repressing Ghd7. *Plant Science* 252**,** 133-143.

Yang, Y.-Y., Shan, W., Kuang, J.-F., Chen, J.-Y., and Lu, W.-J. (2020). Four HD-ZIPs are involved in banana fruit ripening by activating the transcription of ethylene biosynthetic and cell wall-modifying genes. *Plant Cell Reports* 39**,** 351-362.

Yang, Y., Luang, S., Harris, J., Riboni, M., Li, Y., Bazanova, N., Hrmova, M., Haefele, S., Kovalchuk, N., and Lopato, S. (2018). Overexpression of the class I homeodomain transcription factor Ta HDZ ipI‐5 increases drought and frost tolerance in transgenic wheat. *Plant biotechnology journal* 16**,** 1227-1240.

Zhang, R.-X., Zhu, W.-C., Cheng, G.-X., Yu, Y.-N., Li, Q.-H., Ul Haq, S., Said, F., and Gong, Z.-H. (2020). A novel gene, CaATHB-12, negatively regulates fruit carotenoid content under cold stress in Capsicum annuum. *Food & nutrition research* 64**,** 10.29219/fnr.v64.3729.

Zhang, Z., Zhu, R., Ji, X., Li, H.J., Lv, H., and Zhang, H.Y. (2021). Genome-Wide Characterization and Expression Analysis of the HD-ZIP Gene Family in Response to Salt Stress in Pepper. *International Journal of Genomics* 2021**,** 8105124.

Zhao, P., Cui, R., Xu, P., Wu, J., Mao, J.L., Chen, Y., Zhou, C.Z., Yu, L.H., and Xiang, C.B. (2017). ATHB17 enhances stress tolerance by coordinating photosynthesis associated nuclear gene and ATSIG5 expression in response to abiotic stress. *Sci Rep* 7**,** 45492.

Zhao, S., Wang, H., Jia, X., Gao, H., Mao, K., and Ma, F. (2021). The HD‐Zip I transcription factor MdHB7‐like confers tolerance to salinity in transgenic apple (Malus domestica). *Physiologia Plantarum* 172**,** 1452-1464.

Zhao, Y., Ma, Q., Jin, X., Peng, X., Liu, J., Deng, L., Yan, H., Sheng, L., Jiang, H., and Cheng, B. (2014). A novel maize homeodomain-leucine zipper (HD-Zip) I gene, Zmhdz10, positively regulates drought and salt tolerance in both rice and Arabidopsis. *Plant Cell Physiol* 55**,** 1142-1156.

Zhu, Z., Sun, B., Xu, X., Chen, H., Zou, L., Chen, G., Cao, B., Chen, C., and Lei, J. (2016). Overexpression of AtEDT1/HDG11 in Chinese kale (Brassica oleracea var. alboglabra) enhances drought and osmotic stress tolerance. *Frontiers in plant science* 7**,** 1285.
